# Glycan Shape, Motions, and Interactions Explored by NMR Spectroscopy

**DOI:** 10.1021/jacsau.3c00639

**Published:** 2024-01-03

**Authors:** Göran Widmalm

**Affiliations:** Department of Organic Chemistry, Arrhenius Laboratory, Stockholm University, S-106 91 Stockholm, Sweden

**Keywords:** carbohydrate, conformation, coupling constant, molecular dynamics, recognition, relaxation, simulation

## Abstract

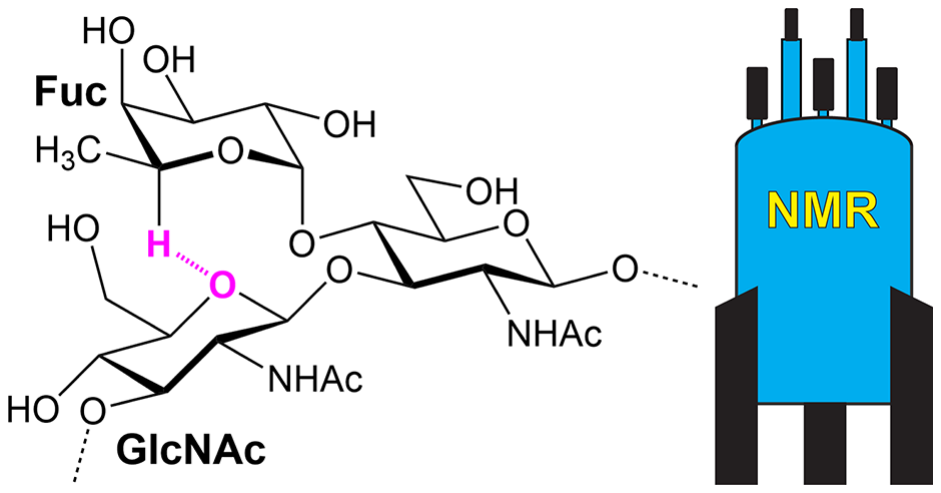

Glycans in the form of oligosaccharides, polysaccharides, and glycoconjugates are ubiquitous in nature, and their structures range from linear assemblies to highly branched and decorated constructs. Solution state NMR spectroscopy facilitates elucidation of preferred conformations and shapes of the saccharides, motions, and dynamic aspects related to processes over time as well as the study of transient interactions with proteins. Identification of intermolecular networks at the atomic level of detail in recognition events by carbohydrate-binding proteins known as lectins, unraveling interactions with antibodies, and revealing substrate scope and action of glycosyl transferases employed for synthesis of oligo- and polysaccharides may efficiently be analyzed by NMR spectroscopy. By utilizing NMR active nuclei present in glycans and derivatives thereof, including isotopically enriched compounds, highly detailed information can be obtained by the experiments. Subsequent analysis may be aided by quantum chemical calculations of NMR parameters, machine learning-based methodologies and artificial intelligence. Interpretation of the results from NMR experiments can be complemented by extensive molecular dynamics simulations to obtain three-dimensional dynamic models, thereby clarifying molecular recognition processes involving the glycans.

## Introduction

**Figure 1:**
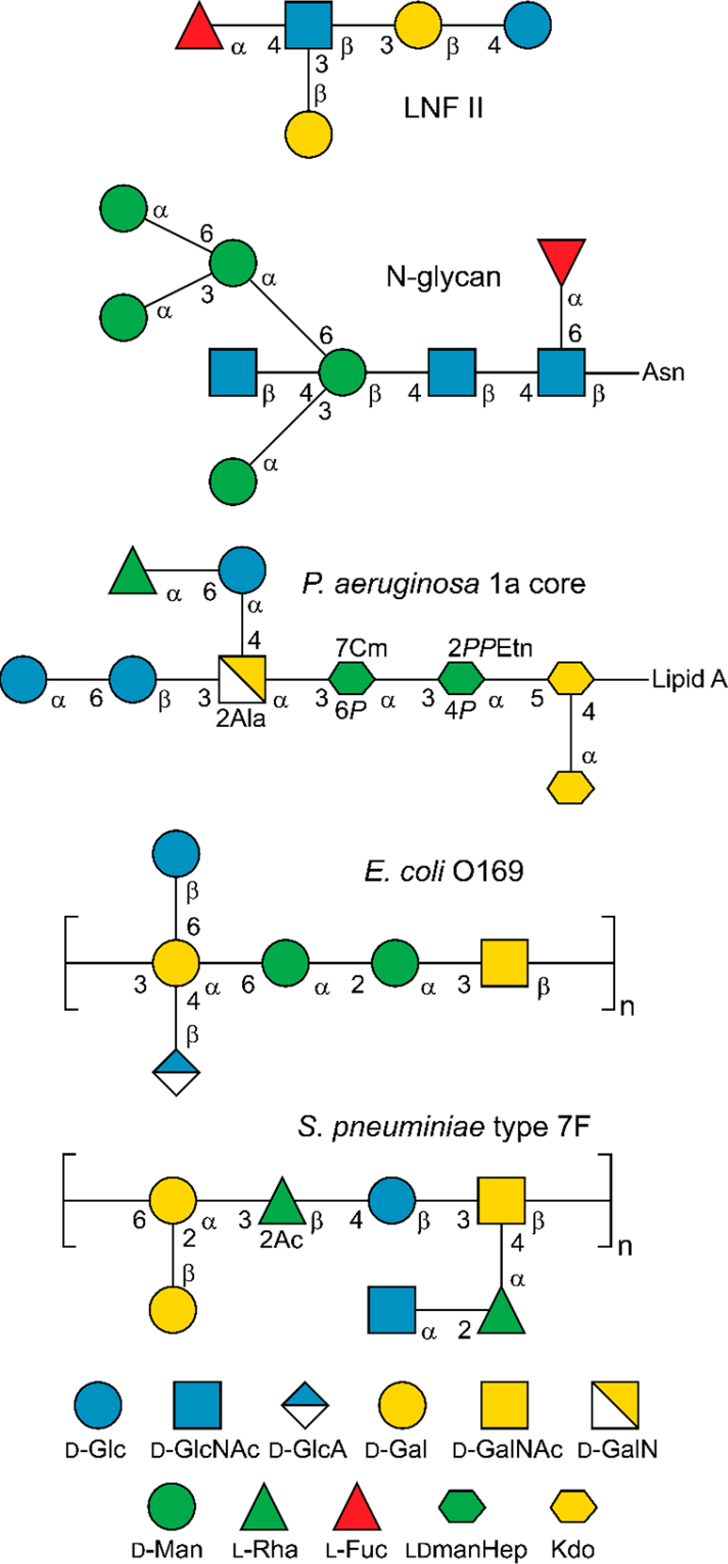
Glycan structures schematically represented using the SNFG format (https://www.ncbi.nlm.nih.gov/glycans/snfg.html#nomn)^134^ drawn by GlycanBuilder2.^135^ From top to bottom: human milk oligosaccharide lacto-*N*-fucopentaose II, N-linked oligosaccharide, core 1a from *Pseudomonas aeruginosa* lipopolysaccharide (LPS),^136^ O-antigen repeating unit of the LPS from *Escherichia coli* O169,^137^ and repeating unit of the capsular polysaccharide from *Streptococcus pneumoniae* type 7F;^138^ SNFG symbols represent specific monosaccharides.

Glycans constitute together with proteins and lipids the core of molecules central to a vast number of biological processes.^1^ The monosaccharide moieties linked together to form biologically active molecules are present as oligosaccharides, e.g., human milk oligosaccharides (Figure 1) that function as soluble decoy receptors blocking the attachment of opportunistic pathogens to the mucosal surface in infants. Polysaccharides such as hyaluronan, which contains disaccharide repeating units, can contain more than 20,000 sugar residues, resulting in a molecular mass of several million daltons and are a component of soft connective tissues where they have unique rheological properties. Glycoconjugates in which the oligo- and polysaccharides are linked to proteins or lipids constitute a major part of the carbohydrate-containing compounds that are found in prokaryotes and eukaryotes. More than half of all proteins in nature have been estimated to be glycosylated^2^ where the glycans, inter alia, shield the protein surface from antibody-recognition, modulate protein aggregation, act as a shield to proteolysis, mask epitopes, and assist in immune evasion or protect tissue surfaces from microbial attachment. The glycan portion is commonly either N-linked (Figure 1) or O-linked to the polypeptide chain, but glycans may also be C-linked via tryptophan residues. Carbohydrates conjugated to lipids are found as glycosphingolipids and glycoglycerolipids. Lipopolysaccharides (LPS) in Gram-negative bacteria are constituents of the outer leaflet in the outer membrane and can have an oligomeric core structure (Figure 1) linked to Lipid A, which contains 4–7 lipid chains. The O-antigen polysaccharides in the LPS, extending from the core region, contain repeating units with 2–7 sugar residues (Figure 1) replicated 10–100 times. Capsular polysaccharides (CPS) (Figure 1) as well as exopolysaccharides, which also contain repeating units, have a molecular mass on the order of megadaltons.

The presence or absence of specific glycan structures is key to health and disease. Mumps virus (MuV) is an aerosol-transmitted human pathogen affecting salivary glands and several other organs, and it infects the central nervous system causing meningitis and encephalitis.^3^ The MuV attachment protein hemagglutinin-neuraminidase (MuV-HN) was via a glycan-binding assay shown to preferentially use a trisaccharide containing an α-2,3-linked sialic acid in unbranched sugar chains as a receptor, whereas the structures having a terminal α-2,6-linked sialic acid were not favored. A crystal structure of MuV-HN in complex with the trisaccharide 3′-sialyllactose revealed that not only was the sialic acid essential, but also the third sugar was involved in receptor binding. Moreover, whereas 3′-sialyllactose can be a terminal structure in glycosphingolipids the closely related 3′-sialyllactosamine may constitute a component in glycoproteins. Thus, MuV may use either of the glycan structures for binding and infection. The ganglioside GM1a having a branched sialic acid-containing pentasaccharide as its glycan portion of the molecule has been shown to function as a coreceptor and attachment factor for dengue virus infection, underscoring the importance of sialic acid-recognition as receptors in glycan-protein based interactions and infections. Interestingly, in binding of the virus to the ganglioside the movement of the virus on the cell surface is accelerated, which may be due to a reduction in rigidity of cellular rafts.^4^ In host–pathogen interactions, including glycan mimicry by bacteria,^5^ the discrimination of self/nonself identity, relying on structure and glycosylation patterns, is essential in resistance to pathogens and to combat infection.^6^

Aberrant glycosylation is a classic hallmark of malignant transformation and antigenic determinants can be used as cancer biomarkers. The structural changes include specific glycan epitopes in N- and O-linked glycoproteins.^7,8^ In treatment of cancers, autoimmune/chronic inflammatory diseases and infection, antibodies of the Immunoglobulin G (IgG) isotype are of paramount importance.^9^ Sugars attached to the IgG Fc (fragment crystallizable) region, i.e., the tail region of the antibody that interacts with cell surface receptors, play a decisive role for anti-inflammatory activity and glycoforms having terminal sialic acid residues are important for interaction with Fc-receptors.^10^ Glycans on IgG antibodies that lack fucose residues in the N-glycan structure can be responsible for enhancing Dengue virus infection and pathology in hosts,^11^ emphasizing the significance of glycan structures on the Fc-domain. Development of therapeutic monoclonal and biosimilar antibodies shows great promise for treatment of various diseases, though heterogeneity in the glycosylation of IgG molecules requires attention for the use of monoclonal antibodies in drug therapy, in order to minimize side-effects due to immunogenic glycans. Furthermore, the already approved clinical use of therapeutic antibodies with low levels of Fc core fucose^12^ underscores the complexity of glycan structures in biological systems and for treatment of disease.

In order to understand structural characteristics of glycans in relation to cell–cell interactions^13^ and how information transfer in glycan-based communications takes place,^14^ a thorough understanding of conformation, dynamics, and recognition of carbohydrates and glycoconjugates is a prerequisite. To this end, solution-state NMR spectroscopy is well suited to unravel many aspects of shape, motions and interactions at an atomic level of detail.

## Shape (Conformation) and Motions (Dynamics)

The commonly occurring ring forms of monosaccharides are furanoses and pyranoses and whereas the former generally are to be described by a conformational equilibrium between a few interconverting twist and envelope forms^15,16^ the latter can in many cases be, to a first approximation, regarded as existing in a well-defined single chair conformation, ^4^*C*_1_ for hexose sugars having the d absolute configuration and ^1^*C*_4_ for hexose sugars having the l absolute configuration. The orientations of hydroxyl groups of a pyranose sugar, in a specified conformation, define its shape and also the ordering of water molecules around it, which can be viewed by spatial distribution functions that give insight into how solvent is arranged around the sugar molecule.

**Scheme 1:**
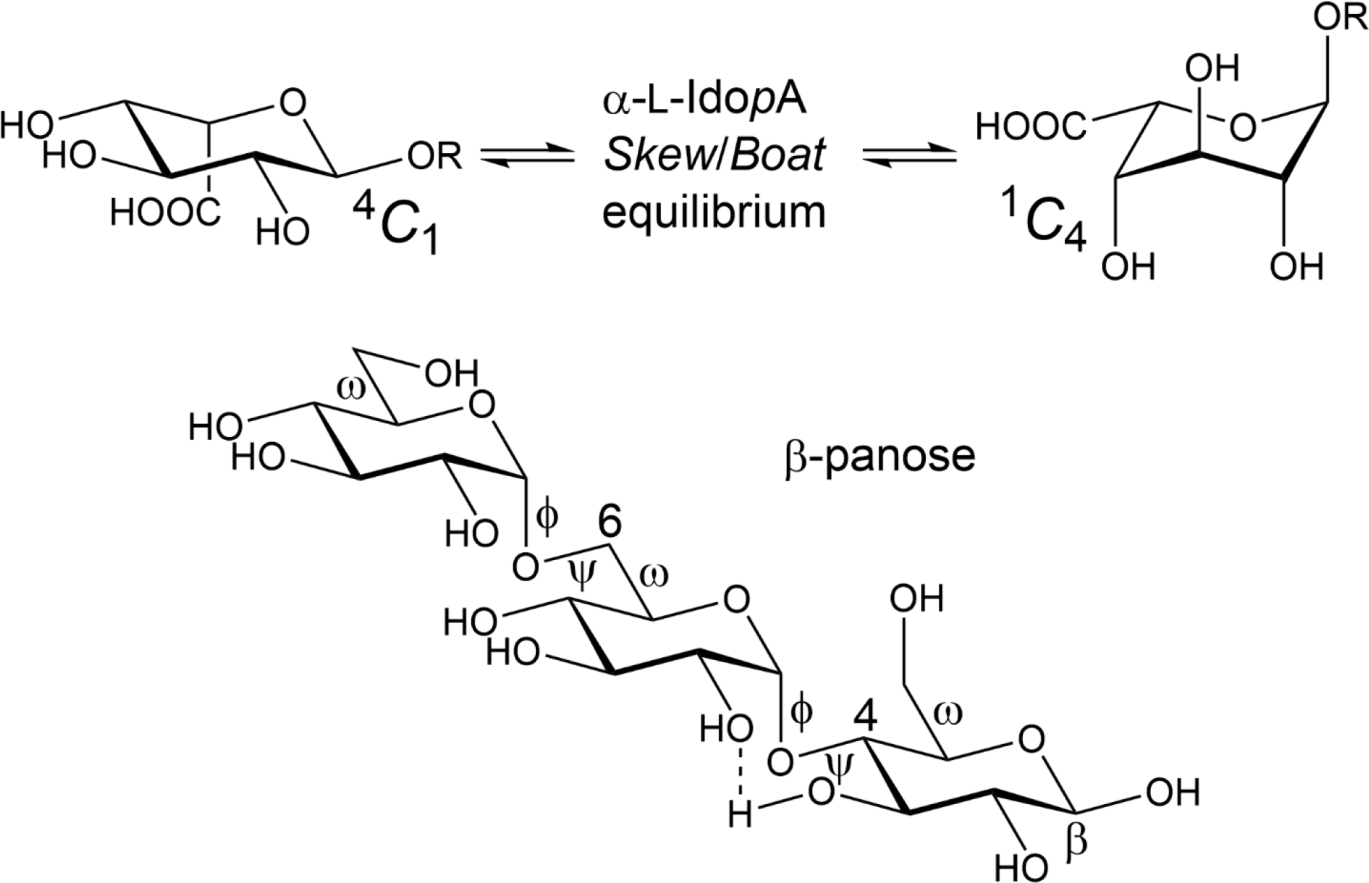
(Top) Conformational equilibrium of α-l-idopyranosiduronic acid (R = H) or as a glycoside (R = Me or aglycone in general) of a three-state model, e.g., ^4^*C*_1_ ⇋ ^2^*S*_O_/*B*_3,O_ ⇋ ^1^*C*_4_; (bottom) trisaccharide β-panose (β-anomeric configuration at the reducing end residue), α-d-Glc*p*-(1 → 6)-α-d-Glc*p*-(1 → 4)-β-d-Glc*p* illustrating the glycosidic torsion angles ϕ and ψ at the α-(1 → 4)-linkage and ϕ, ψ and ω at the α-(1 → 6)-linkage. The conformation of the exocyclic hydroxymethyl groups defined by the ω torsion angle have been drawn in the three staggered rotamers populated to different extent depending on stereochemistry and stereoelectronic effects. Potential inter-residual hydrogen bonding between O2’ and HO3 is shown by a dashed line.

However, some stereoisomers of pyranoses exist in more than one of the 38 canonical ring forms^17^ and need to be described by additional conformations in an equilibrium,^18^ e.g., idose and derivatives thereof such as the uronic acid IdoA (Scheme 1). Even the small structural difference between the reducing d-idose and its methyl glycoside results in conformational changes with different relative populations in a two-state equilibrium model, both for the α-anomeric and the β-anomeric forms. Moreover, the ring conformational equilibrium for methyl α-l-idopyranosiduronic acid is pH-dependent. The conformational preference and equilibrium of idopyranose was recently revisited by Haasnoot et al.^19^ and in addition to the two chair conformations of the pyranose ring, ^4^*C*_1_ and ^1^*C*_4_, which exhibit the same ring pucker regardless of the ring substituent pattern, also the skew ring conformation ^2^*S*_O_ is populated to a significant extent. When the equilibrium was modeled by three conformational states based on NMR proton–proton coupling constants it was noted that ^3^*J*_H1,H2_ and ^3^*J*_H4,H5_ deviated between calculated and observed *J* couplings for these endocyclic proton pairs. Importantly, by considering the pseudorotation phase angle *P*_2_ of the skew/boat equilibrium, along the equator of the Cremer–Pople (CP) sphere (often viewed as a two-dimensional Mercator projection), consistency of the *J* couplings were observed for conformations halfway between ^2^*S*_O_ and *B*_3,O_. Importantly, the population of the ^2^*S*_O_/*B*_3,O_ conformer is maintained to ∼30% over a wide variety of experimental conditions for *ido*-pyranoses and as their glycosides while ^4^*C*_1_ and ^1^*C*_4_ conformations vary more or less *inversely* proportional to each other. Glycosides of α-l-idopyranosiduronic acid with bulkier aglycones compared to the methyl glycoside have a preference for the (quasi-)equatorial orientation of the C1–O1 bond, i.e., the ^2^*S*_O_/*B*_3,O_ and ^4^*C*_1_ conformations. Furthermore, in 2-*O*-sulfo-α-l-idopyranosiduronic acid glycosides the *B*_3,O_ conformation positions the charged sulfo- and carboxylate groups farthest apart. Taken together, the substitution pattern of the idopyranose ring and/or specific sulfation pattern(s) of adjacent sugar residues govern the skew/boat equilibrium adapted by idopyranose residues, findings that are in accord with NMR studies of heparan sulfate-related hexasaccharides and molecular dynamics (MD) simulations thereof^20^ as well as MD simulations of constituent monosaccharides.^21^

Cleavage of heparan sulfate (HS), a sulfated glycosaminoglycan, by human heparanase releases growth factors and overexpression of the protein is linked to inflammation and cancer metastasis. The understanding that 2-*O*-sulfo-α-l-idopyranosiduronic acid (IdoA2S) is inherently flexible facilitates insight into how a cationic highly charged platinum complex (TriplantinPC) interacts with a highly sulfated pentasaccharide (Fondaparinux), which has as one of its monosaccharides a IdoA2S residue.^22^ In the pentasaccharide, the conformational equilibrium of Ido2S shows canonical ring forms ^2^*S*_O_:^1^*C*_4_ in a relative ratio of 65:35, whereas when complexed with TriplantinPC the ratio is 25:75, thus favoring the inverted chair conformation. Counterion condensation effects may also contribute to the altered conformational equilibrium upon binding of TriplantinPC to a HS model, and consequently in binding to glycosaminoglycan polyelectrolytes. The importance of being able to tune conformational equilibria and to form biologically active complexes can be seen from the fact that TriplantinPC inhibits HS cleavage in human cells, thereby preventing growth factor binding and signaling.

**Figure 2:**
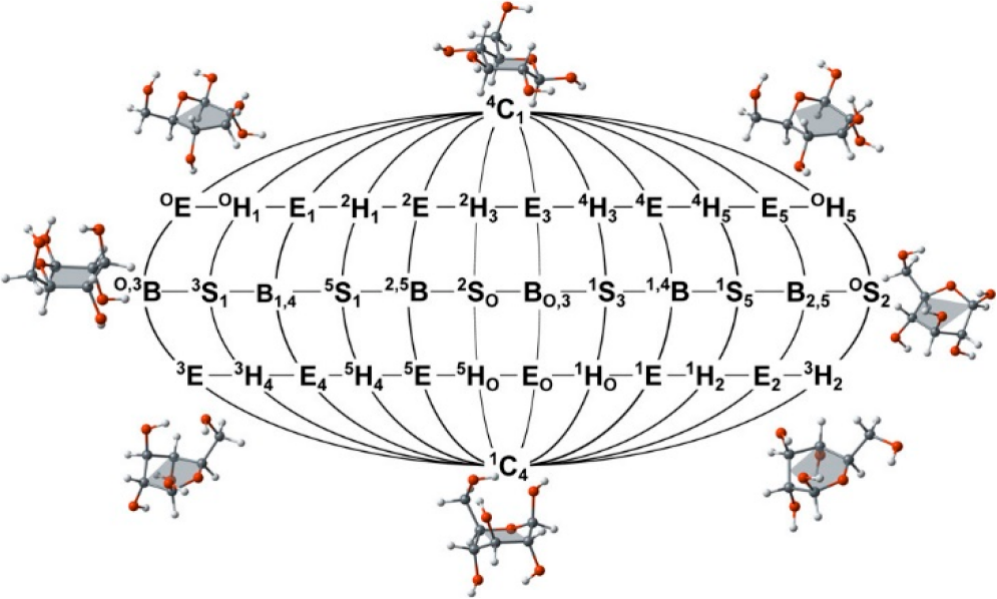
Two-dimensional projection of the Cremer–Pople (CP) sphere shows the 38 canonical puckering designations. The letter designates the type of pucker (chair, half-chair, envelope, skew, or boat), and the conformations on the outer ring are illustrated using β-glucose. Reproduced from ref (23). Copyright 2014 American Chemical Society.

**Figure 3:**
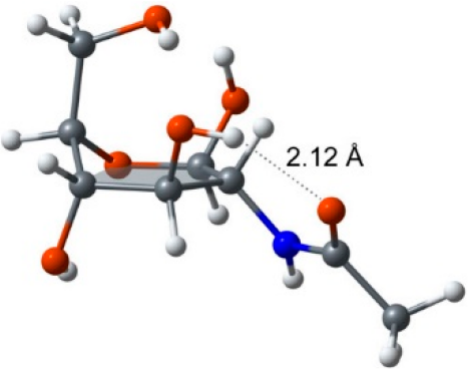
β-d-Glc*p*NAc in the envelope conformation ^5^*E* calculated to be a local energy minimum. Not only can hydrogen bonding take place between the carbonyl oxygen of the *N*-acetyl group and HO3 (dotted line) but having C5 positioned above the plane of the ^5^*E* conformation hydrogen bonding is also possible between the hydroxymethyl group (above the five-atom plane) and HO1 or HO3. Reproduced from ref (23). Copyright 2014 American Chemical Society.

To investigate “how sugars pucker,” Mayes et al.^23^ performed quantum chemical (QM) calculations on d-hexopyranoses commonly being part of glycans of biological importance. A large number of conformations for each monosaccharide was initially generated based on the 38 canonical ring forms and rotamers of exocyclic torsions (hydroxyl groups, and when present hydroxymethyl and *N*-acetyl groups). Subsequent QM-based geometry optimization at different levels of theory was employed to identify local potential energy minima and transition state structures on the itinerary of puckering interconversion pathways (Figure 2). In five sugars having the pyranoid ring form (β-d-Xyl*p*, α-d-Glc*p*, β-d-Glc*p*, β-d-Man*p*, and β-d-Glc*p*NAc) and chosen to have at least four equatorially oriented groups, these were present in the ^4^*C*_1_ conformation. The computational approach identified pathways for the interconversion from the low energy ^4^*C*_1_ conformation (North pole) via different skew (*S*) and boat (*B*) conformations (along the equator) to the ^1^*C*_4_ conformation (South pole), all of which shows a higher free energy for the latter inverted conformation of the hexopyranoses having the d absolute configuration. The barrier heights (Δ*H*^‡^) for conversion from the ^4^*C*_1_ conformation to local energy minima of *S* and *B* conformations are generally ∼8 kcal·mol^–1^ and higher (for β-d-Glc*p* and β-d-Glc*p*NAc Δ*H*^‡^ ∼6 kcal·mol^–1^ to the *B*_3,O_ conformation). In comparing the free energy (Δ*G*) to the ^4^*C*_1_ conformation of the hexopyranoses the relative difference of puckered conformations is ∼3–4 kcal·mol^–1^, where β-d-Glc*p*NAc shows the lowest relative Δ*G* of ∼3 kcal·mol^–1^ among pucker conformations being both *S* and *B*. The ^1^*C*_4_ conformation is on the order of ∼6 kcal·mol^–1^ higher than the ^4^*C*_1_ conformation, except for β-d-Xyl*p* that is only ∼2 kcal·mol^–1^ higher. These results can be compared to Δ*H*^‡^ and relative Δ*G* for the ^4^*C*_1_ ⇋ ^1^*C*_4_ conformational equilibrium for the pyranose ring form of *N*-acetyl-d-allosamine, the C3 epimer of β-d-Glc*p*NAc. From enhanced-sampling free energy metadynamics MD simulations of α-d-All*p*NAc and β-d-All*p*NAc^16^ Δ*H*^‡^ barriers were ∼10 kcal·mol^–1^ and higher, i.e., similar to those for hexopyranoses computed using QM-based methods (vide supra), and Δ*G* was ∼6 kcal·mol^–1^ higher for the inverted chair conformation, slightly higher for the anomeric forms of the latter monosaccharide having the *allo*-configuration. Thus, although pucker conformations and even inverted chair conformations are accessible for hexopyranoses from an energetic point of view, as indicated from computational studies, they may due to the low populations be elusive to identify using experimental NMR techniques. However, in the thermodynamic and kinetic landscape of β-d-Glc*p*NAc the exocyclic *N*-acetyl group can participate in hydrogen bonding thereby stabilizing the structure, such as in the envelope conformation ^5^*E* (Figure 3). This may have implications for vicinally disubstituted residues, such as in 3,4-di-*O*-glycosylated β-d-Glc*p*NAc, in that nonchair conformations may still be present since vicinal proton–proton scalar NMR coupling constants differ significantly from nonsubstituted β-d-Glc*p*NAc residues,^24^ findings that suggest intrinsic monosaccharide flexibility to be of importance in defining the shape of glycans.

In molecular modeling of carbohydrates, additive or nonpolarizable force fields based on Coulomb’s law to treat electrostatic interactions, in which partial atomic charges are static or fixed whereby the electrostatic energy of the system is simply treated as the sum of all individual atom–atom Coulombic interactions, are commonly used to explore conformational space and shape of the molecules.^25^ However, the shortcoming of having fixed partial atomic charges without explicit treatment of electronic polarizability in order to be able to adjust in response to the local electric field is a limitation. A remedy for this is, among others, the classical Drude oscillator model, also known as the “charge-on-spring model”, in which explicit polarization is implemented by attaching a charged auxiliary particle with a harmonic spring to the core of the polarizable atom, thereby allowing for the atomic dipoles to adjust in response to the surrounding electric field. However, sampling conformational space and investigation of ring puckering for d-hexopyranoses show that the CARMM36 additive force field tends to overstabilize the ^4^*C*_1_ conformation, whereas the Drude polarizable carbohydrate force field results in flexible ring systems, more readily accessible skew and boat conformations, lower free energy barriers, and oversampling of the ^1^*C*_4_ conformation for some of the monosaccharides.^26^ The inverted ^1^*C*_4_ conformation for α-d-mannopyranose was in these MD simulations present to an extent of ∼80%, whereas in a study of α-linked d-mannopyranose disaccharides using the Drude polarizable force field a wider conformational sampling at glycosidic linkages was evident, without any oversampling of inverted chairs.^27^ Even though sampling of conformational space by MD simulations using the Drude model is ∼4-fold slower than the additive CHARMM force field, and ∼8-fold slower than using a hydrogen mass repartitioning (HMR) approach by increasing the mass of hydrogen by distributing a heavy atom mass to hydrogen atom(s) attached to the heavy atom,^28^ the modeling is performed in a more realistic way, for glycans per se but in particular for understanding and unveiling the structural basis of ligand recognition and specificity in carbohydrate–protein interactions, which calls for further developments of the Drude polarizable force fields. Moreover, the choice of water model for MD simulations may be crucial for the outcome and the resulting conclusions that can be made,^29−31^ and so can the absence or presence of small partial atomic charges on aliphatic protons of rhamnosyl residues be in modeling of polysaccharides.^32^

In oligo- or polysaccharides, the monosaccharides are joined together via glycosidic linkages in which two degrees of freedom, ϕ and ψ, describe the available conformational space related to the glycosyloxylated carbon atom, i.e., when a secondary alcohol is glycosylated (Scheme 1). In cases when a primary alcohol is glycosylated by a sugar residue an additional degree of freedom is present, e.g., in (1 → 6)-linked hexopyranosides where the torsion angle ω is related to the exocyclic hydroxymethyl group or in (2 → 9)-linked sialic acids. The definitions commonly used in solution state NMR spectroscopy of aldoses in a disaccharide are given by ϕ = H1′–C1′–O*n*–C*n* and ψ = C1′–O*n*–C*n*–H*n*, though other torsional angle definitions describing conformational space are in use based on IUPAC definitions or employed in studies related X-ray crystallography; *n* is the substitution position of the sugar residue at the reducing end and the prime refers to atoms in the terminal glycosyl entity, whereas in ketoses such as sialic acids the former torsion angle is defined by ϕ = C1′–C2′–O*n*–C*n*^33^ or ϕ = O6′–C2′–O*n*–C*n*.^34^ Furthermore, in hexopyranoses the exocyclic torsion angle ω = O5–C5–C6–O6 is the usual definition, although other definitions can be used to describe the torsion angle.^35^ The conformational space sampled by the glycans can be subdivided into conformational states that are populated to various degrees based on the relative orientations of the sugar residues to each other, viz., a syn-state in which the protons at the carbon atoms at the glycosidic linkage are close to each other and on the same side of a plane orthogonal to the C1′-H1’ bond of the sugar residue at the glycosidic linkage. In general, the major conformation at a glycosidic linkage has the syn-state, and the ϕ torsion angle populates to a large extent an exoanomeric conformation in which ϕ ≈ +40° for β-d- and α-l-hexopyranosides and ϕ ≈ –40° for α-d- or β-l-hexopyranosides, but a nonexoanomeric conformation may also be populated, where the ϕ torsion angle is shifted such that O5′-C1′-O*n*-C*n* becomes antiperiplanar, anticipated to have a larger contribution for sugar residues that have the *manno*-configuration; i.e., the hydroxyl group at C2 is then oriented axially in the ^4^*C*_1_ ring conformation of d-mannose or in the ^1^*C*_4_ ring conformation of l-rhamnose (6-deoxy-l-mannose). Additional conformational states accessible are those in which an anti-ϕ and anti-ψ are populated having the torsion angle describing an antiperiplanar conformation. Besides the syn-state with an exoanomeric conformation as the major conformation the additional conformational states together describe the shape of the saccharides at each glycosidic linkage of an oligo- or polysaccharide. Thus, the three-dimensional (3D) structure and shape of a glycan molecule can, based on this knowledge, be employed to rapidly generate molecular models using software dedicated to this end, such as CarbBuilder,^36^ or web-based tools, e.g., Glycan Reader & Modeler in CHARMM-GUI^37^ or GLYCAM-Web (https://glycam.org). The importance of being able to efficiently obtain structures of a glycan under investigation is key to interpretation of NMR spectral data and to identify epitopes that may be essential for the molecular interactions between carbohydrates and proteins, whether antibodies, lectins from plants utilized to assay interactions or selectins, of which the latter are cell-surface lectins that mediate adhesion of white blood-cells to endothelial cells by recognizing fucosylated or sialylated glycoproteins.

**Figure 4:**
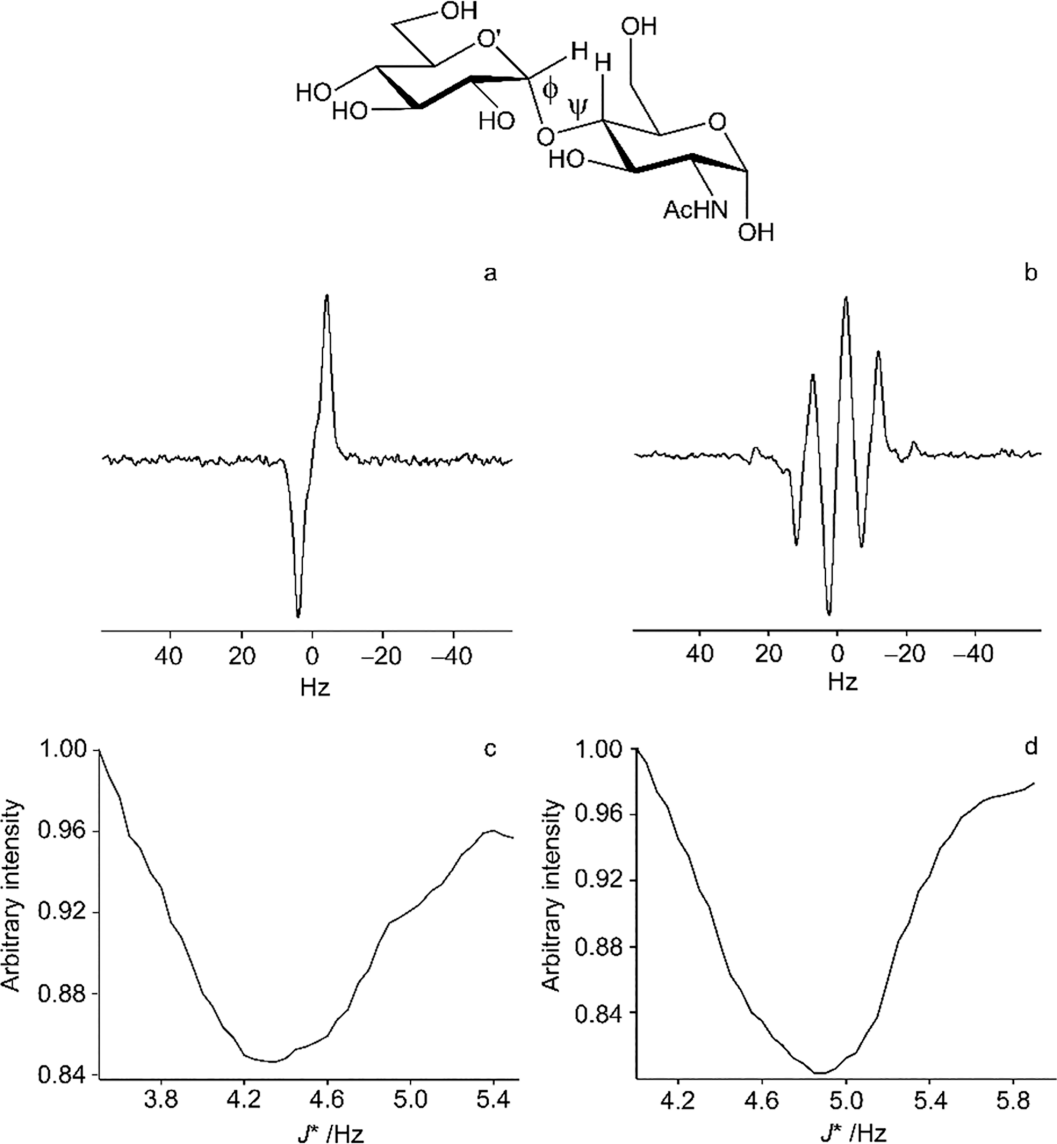
(Top) Schematic representation of α-d-Glc*p*-(1 → 4)-α-d-Glc*p*NAc. Results from a one-dimensional long-range (1DLR) NMR experiment^139^ on the disaccharide at 300 K in D_2_O: (a) H1’ resonance after selective excitation of C4; (b) H4 resonance after selective excitation of C1’; (c) minimum of the integral plot employing *J*-doubling in the frequency domain on the multiplet from (a) is used to identify the value of the coupling constant between H1’ and C4, viz., ^3^*J*_H1′,C4_ = 4.34 Hz, and (d) the same procedure applied to the multiplet from (b) to identify the *J* value between C1’ and H4, viz., ^3^*J*_C1′,H4_ = 4.85 Hz. The transglycosidic coupling constants for α-d-Glc*p*-(1 → 4)-β-d-Glc*p*NAc with the β-anomeric form at the reducing end residue are closely similar to ^3^*J*_H1′,C4_ = 4.18 Hz and ^3^*J*_C1′,H4_ = 4.75 Hz and so are the coupling constants for α-d-Gal*p*-(1 → 4)-α-d-Glc*p*NAc having ^3^*J*_H1′,C4_ = 4.40 Hz and ^3^*J*_C1′,H4_ = 5.07 Hz and α-d-Gal*p*-(1 → 4)-β-d-Glc*p*NAc with ^3^*J*_H1′,C4_ = 4.27 Hz and ^3^*J*_C1′,H4_ = 4.95 Hz, indicating similar conformational averaging among the disaccharides (T. Angles d’Ortoli and G. Widmalm, unpublished results). The span of the three-bond heteronuclear transglycosidic coupling constants from Karplus-type relationships is ∼7 Hz.^140^

Classical ^1^H,^1^H-NOESY NMR experiments can give short-range distance information across glycosidic linkages in polysaccharides, and 1D ^1^H,^1^H-T-ROESY or 1D ^1^H,^1^H-ROESY experiments are well-suited to efficiently reveal this information in oligosaccharides, which for branched structures may give additional information to generate a three-dimensional model based on distance information between sugar residues. However, the NOE-based information may be limited and the conformational dependence of transglycosidic three-bond coupling constants (Figure 4), interpreted via Karplus-type relationships, are quite sensitive to changes in torsion angles at the glycosidic linkage. By complementing measured ^3^*J*_CH_ coupling constants^38^ with homonuclear ^3^*J*_CC_ coupling constants, which may be determined at natural abundance, preferentially using NMR spectrometers with cryogenic probes of high sensitivity, several conformationally dependent coupling constants can be obtained. Common schemes to label oligosaccharides employing ^13^C isotopologues, which only differ in isotopic composition, include single isotope enrichments, which from a spectral interpretation point of view makes the analysis straightforward and NMR spin-simulation approaches containing both ^1^H and ^13^C nuclei can be performed, though the approach may become laborious and tedious with respect to the chemical or enzymatic synthesis of singly labeled compounds produced one-at-a-time. In doubly labeled compounds one approach is to use a [1,2-^13^C_2_]-labeling from which, inter alia, transglycosidic ^3^*J*_CC_ coupling constants related to both ϕ and ψ can be obtained, whereas labeling adjacent to the glycosidic linkage, i.e., next to the glycosidic oxygen atom at positions C1’ and C4 or C1” and C6’ in panose (atom labeling in sugar residues usually begins from the reducing end residue being nonprimed, followed by a prime in the subsequent residue, a double prime etc. in small oligosaccharides), will give the complete set of three-bond coupling constants across the glycosidic linkage. Conformationally dependent ^1^*J*_CH_, ^1^*J*_CC_, ^2^*J*_CH_, ^2^*J*_CC_, and ^*n*^*J*_CC_ coupling constants can be used as a complement and with a set of redundant NMR couplings the conformation or conformational equilibria of oligosaccharides in solution can be investigated using single-state and multistate models to determine rotamer population distributions (*MA’AT* analysis).^39,40^

**Figure 5:**
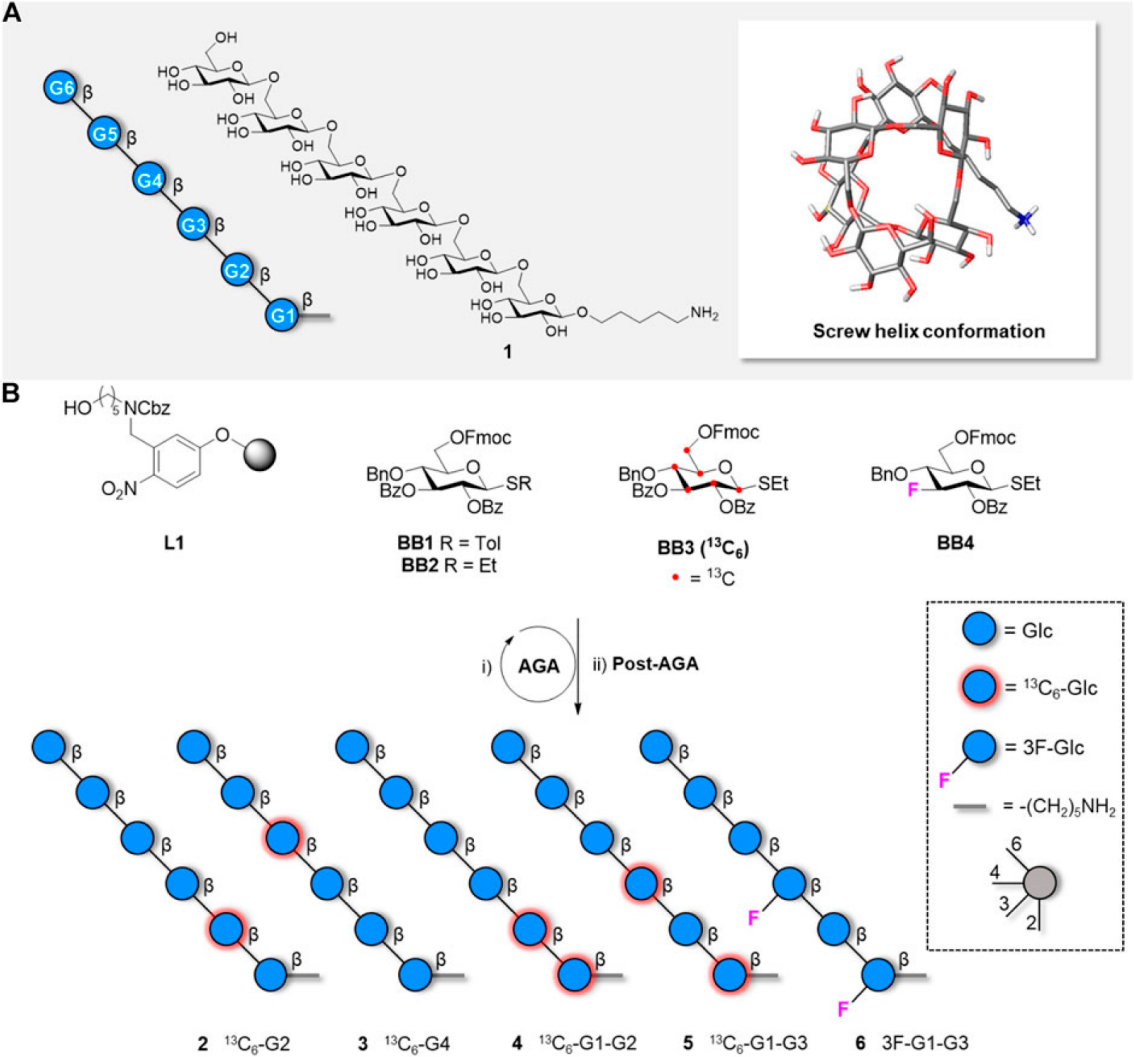
(A) The structure is represented using the SNFG format, drawn as a chemical structure, and presented as a molecular model of the global minimum conformation of hexasaccharide **1** as obtained by molecular dynamics simulations. (B) Synthesis of five hexasaccharide analogues **2**–**6** bearing labeled functionalities in specific positions of the glycan chain. The procedure includes automated glycan synthesis, cleavage from solid support, removal of protecting groups and purification by high-performance liquid chromatography. Reproduced from ref (41). Copyright 2021 Frontiers Media SA.

An alternative, still relying on that only certain nuclei are ^13^C-labeled is to use uniform [UL-^13^C_6_]-labeling of one or more specific sugar residue(s) in an oligosaccharide, a labeling scheme that may give access to a large number of spin–spin coupling constants but also to that other NMR parameters can be measured. The power of automated glycan synthesis was shown for a series of a compounds, viz., a β-(1 → 6)-linked hexaglucoside, ^13^C isotopologues thereof and ^19^F analogues. Subsequent conformational analysis of these oligosaccharides (Figure 5) was based on NMR residual dipolar couplings (RDCs),^41^ an NMR methodology that during the last few decades has been used to complement NOE and *J*-based conformational studies of biomolecules and to provide insight into molecular structure. As the intraring one-bond C–H vectors in a β-d-Glc*p* residue in the ^4^*C*_1_ conformation are all parallel, or nearly so, the specifically derivatized 3-deoxy-3-fluoro-β-d-Glc*p* residues in the hexasaccharide provided valuable experimental RDC data used in the fitting procedure to various model structures, since the orientation of the C3–F vector of the sugar residue is different compared to the intraring C3–H vector. Notably, the molecular conformation in the two alignment media used in the study were different, presumably because the interactions of the solute hexasaccharide with the ionic aromatic cromolyn molecule provided aromatic-glycan interactions leading to preferentially helix-like structures, whereas in the nonionic medium containing C12E5/*n*-hexanol in D_2_O the alignment was weaker, as seen from the deuterium quadrupolar splitting of D_2_O, and it was concluded that an extended conformation was favored in the latter medium where alignment is based primarily on molecular shape. The development of efficient labeling schemes, whether using chemical/enzymatic synthesis, metabolic labeling or enzymatic remodeling to incorporate singly, multiply or uniformly labeled isotopically enriched sugar residues is envisioned to have large impact on our understanding of glycan structure and its advancement will be essential for, in particular, large multiglycosylated proteins.

Metabolic labeling schemes have been devised to obtain ^13^C-labeled mammalian-type oligosaccharides using genetically engineered *Saccharomyces cerevisiae* cells.^42^ The triantennary high-mannose-type oligosaccharide was uniformly labeled using d-[UL-^13^C_6_]glucose as well as selectively labeled employing the six site-specifically ^13^C-enriched glucose isotopomers, thereby facilitating NMR-based conformational studies and aiding resonance assignments. Sparse isotope labeling using d-[UL-^13^C_6_]glucose was applied to the N-terminal domain of the highly glycosylated protein CEACAM1, whereby all glycans plus all alanine residues were isotopically labeled.^43^ The relatively inexpensive procedure employed addition of isotopically labeled glucose to the mammalian cell expression medium corresponding to a 1:1 mixture of labeled and unlabeled glucose, resulting in ^13^C labeling of ∼40% in all sugar residues. The occupancy of three N-glycosylation sites in the CEACAM1-IgV domain carrying oligosaccharides was determined to be ∼50–80% and in conjunction with potentially different glycoforms the NMR resonance assignment of glycan signals becomes a challenging task, though the assignment process can be aided by programs such as CASPER,^24^ which is focused on glycans but also handles glycans linked to the most commonly observed glycan-amino acid entities in glycopeptides and glycoproteins. An alternative approach to isotope labeling of glycans is enzymatic remodeling of N-glycan structures, which was applied to an Fc fragment from an IgG1 antibody using the glycosyltransferase Gnt1 and UDP-[^13^C,^15^N]GlcNAc resulting in an isotope labeled β-(1 → 3)-linkedd-GlcNAc residue in the N-glycan.^44^ By a modified *in vitro* enzymatic conversion scheme using two glycosyltransferases, Gnt1 and Gnt2, in sequence and the isotopically labeled donor molecule, it was possible to install both β-(1 → 3)- and β-(1 → 6)-linkedd-GlcNAc residues onto α-linked mannosyl residues on the N-glycan.

**Figure 6:**
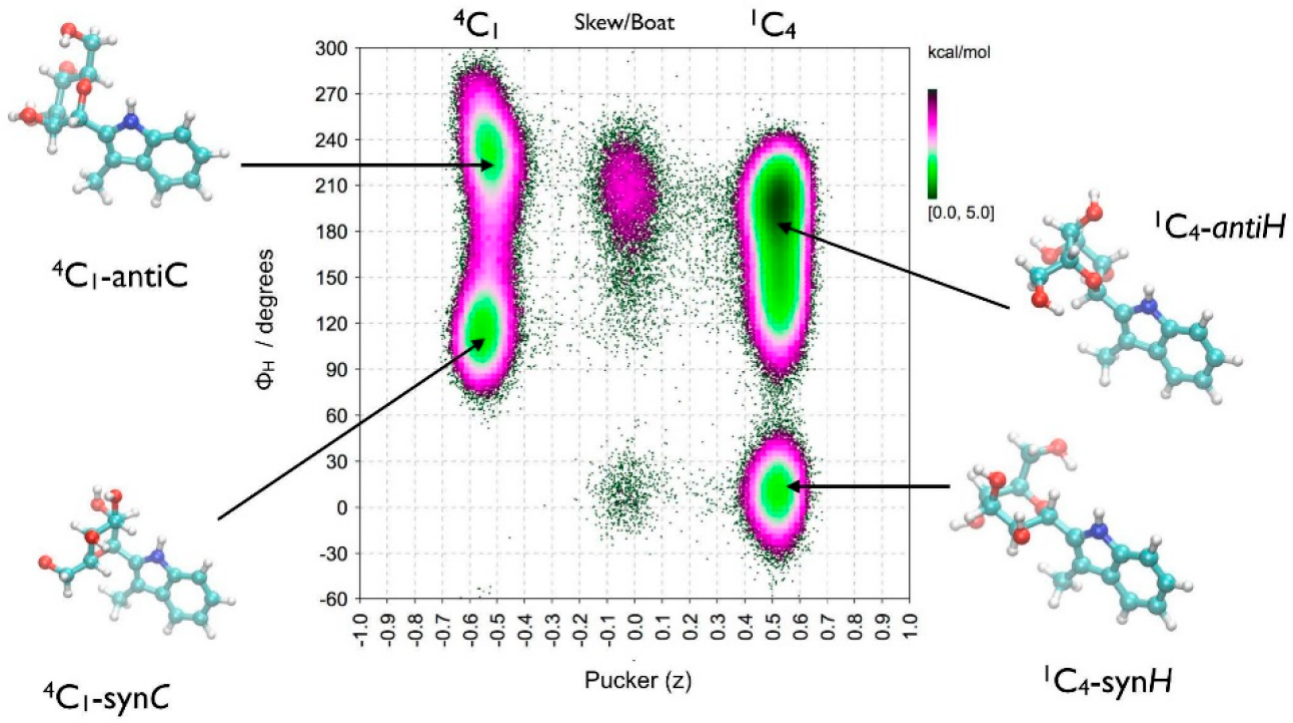
Conformational preferences of α-d-Man*p* linked via a carbon–carbon bond between the anomeric carbon of the sugar and C2 of the 3-methyl-indole as a function of ring puckering coordinate z and glycosidic torsion angle Φ_H_ obtained from MD simulation in explicit solvent at 310 K. Reproduced from ref (46). Copyright 2020 Cell Press.

C-Mannosylation is an unusual type of glycosylated structures in which α-d-Man*p* is linked via a carbon–carbon bond between C1 of the sugar and C2 of the indole entity of tryptophan residues in certain proteins^45^ such as thrombospondin type 1 repeats (TSRs), thereby supporting folding and enhancing stability of the protein.^46^ In the C-mannosylation motif (WxxWxxW), all three tryptophan residues may be glycosylated and substitution on the first two by mannosyl residues increases resistance to thermal degradation of the TSRs. In the three antiparallel strands of the protein bridged by disulfide linkages the tryptophan residues in the first strand intercalate with conserved arginine residues (RxRxR) in the second strand thereby forming a tryptophan-arginine ladder, a structural motif stabilized by cation-π interactions between the side-chains of the amino acids. C-mannosylation is also present in human RNase 2 and NMR studies complemented by MD simulations of peptides isolated from the protein and of native and denatured RNase 2 revealed that the α-d-Man*p* residue exists as an ensemble of conformations of which the ^1^*C*_4_ conformation is the most abundant one, stabilized by a network of hydrogen bonds with the protein resulting in a concomitant reduction of protein dynamics. Besides the internal dynamics of the mannosyl residue the conformational space is also described by its glycosidic torsion angle Φ_H_ for which conformational synC- and antiC-states, related to the orientation of C2 in Man to the nitrogen atom in the indole moiety, are populated in the ^4^*C*_1_ conformation whereas synH- and antiH-states are populated in the ^1^*C*_4_ conformation of the sugar residue (Figure 6). Thus, ring inversion and changes in the preferred orientation of the glycosidic torsion angle are concurrent events. C-Mannosylation is furthermore a structural element in a thrombospondin repeat from the netrin receptor UNC-5 as part of axon pathfinding. Employing a *Drosophila* S2 cell line expressing a single TSR coexpressed with a C-mannosyltransferase in conjunction with a mutation of the enzyme mannose phosphate isomerase resulted in that glucose could not be converted to mannose, which was supplied as uniformly ^13^C-labeled mannose, thereby facilitating a range of NMR experiments to be used to investigate the conformational dynamics of the C-linked mannosyl residues at W5 and W8 of the protein.^47^ The flexibility of the sugar residues attached to the TSR was investigated by heteronuclear ^13^C-relaxation experiments, viz., longitudinal *T*_1_, off-resonance rotating frame *T*_1ρ_, in lieu if transverse *T*_2_, and heteronuclear NOE. The NMR relaxation parameters were analyzed using the model-free dynamical formalism and the mannosyl residues were found to be quite rigid as determined from the high values of the generalized order parameters of the ring carbon atoms, *S*^2^ = 0.85–0.96, where *S*^2^ = 1 corresponds to completely restricted motion and *S*^2^ = 0 to a case where all orientations of the C–H vectors are equally probable. Moreover, the α-d-Man*p* residue covalently linked to W5 of the protein exhibited a short effective correlation time τ_e_ ≈ 60 ps, whereas the 65 amino acid protein construct containing two sugar residues is estimated to have a global reorientational correlation time τ_M_ ≈ 5 ns, based on its molecular mass and a temperature of 298 K (http://nickanthis.com/tools/tau) used in the NMR experiments. Interestingly, the study employed NMR cross-correlated relaxation (CCR) rates between pairs of ^1^H,^13^C dipoles in the mannose residues such that projection angles can be obtained between CH,CH bond vectors. In conjunction with ^3^*J*_HH_ coupling constants the CCR data revealed that the mannosyl residues populate the inverted ^1^*C*_4_ conformation, which also interconverts with canonical boat and skew conformations, viz., the *B*_O,3_ and ^1^*S*_3_ states. These conclusions were further supported by *n*-dimensional NMR experiments including, inter alia, a 4D ^1^H,^13^C,^1^H,^13^C-NOESY-HSQC experiment resolving signal overlap, where the absence of, or only weak H3,H5 NOEs were indicative of the ^1^*C*_4_ conformation. The structural characteristics of the extraordinary C-mannosylation reveal that the hexopyranose α-d-Man*p* residue, which as a monosaccharide may be well represented as having the ^4^*C*_1_ conformation, can under certain conditions pucker into an inverted chair and other canonical ring forms underscoring its inherent flexibility and that this chameleon behavior need to be considered in the analysis of structure and dynamics even of monosaccharides.

**Figure 7:**
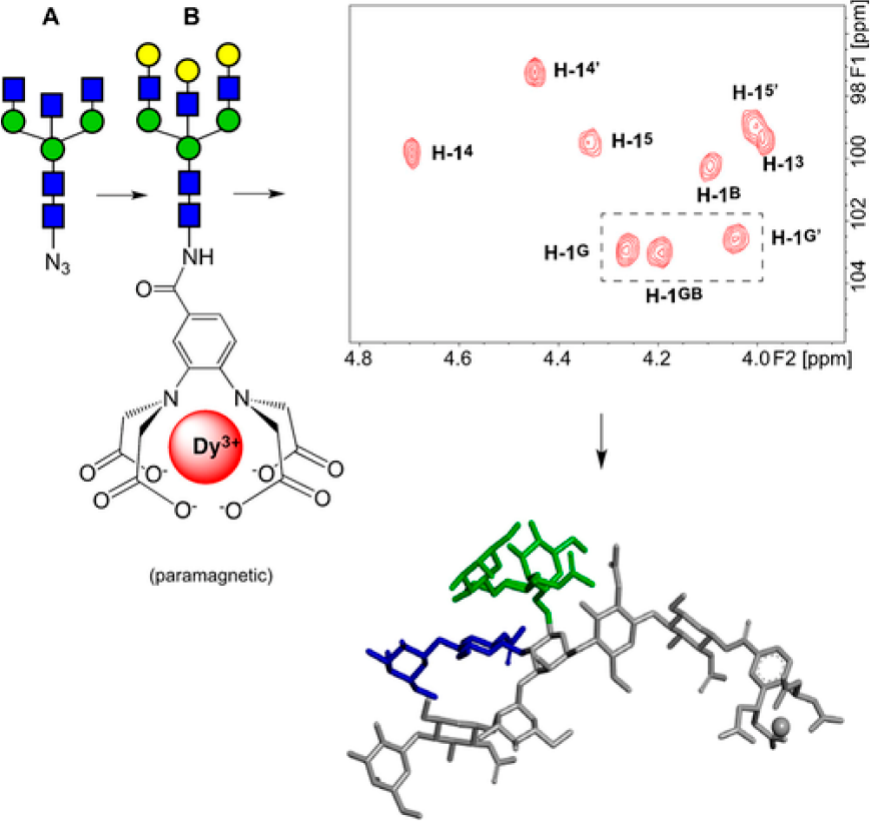
(Upper left) Galactosylation and derivatization with a lanthanide binding tag; (upper right) anomeric region of the ^1^H,^13^C-HSQC NMR spectrum of the undecasaccharide containing paramagnetic dysprosium ion Dy^3+^ complexed with the tag, whereby the cross-peaks of three anomeric protons of the galactosyl residues become resolved (highlighted by the rectangle with dashed lines), in contrast to when the lanthanide tag is complexed with the diamagnetic ion La^3+^ and the cross-peaks overlap at δ_H_/δ_C_ ∼4.34/∼103; (lower right) molecular model of a folded conformation being one of the three main interconverting conformations at the glycosidic linkage of the α-d-Man*p*-(1 → 6)-β-d-Man*p* structural element of the undecasaccharide as derived by analysis of PCS data. Reproduced from ref (48). Copyright 2020 The Authors. Published by Wiley-VCH GmbH.

Glycans are notorious for their limited spectral dispersion where, in particular, ^1^H NMR chemical shifts are observed in a spectral region of only ∼1 ppm, although anomeric protons resonate outside this region and deoxy-sugars having a methylene group as part of the ring system or an exocyclic methyl group may be used as a starting point for resonance assignments, in conjunctions with multidimensional NMR experiments showing correlations to ^13^C nuclei as well as to ^15^N or ^31^P nuclei commonly present in carbohydrates. Spectral overlap is particularly problematic in polysaccharides where only two types of sugar residues are present, say glucose and galactose as in exopolysaccharides produced by some lactic acid bacteria, or in homopolymers, such as mannans, containing only one type of sugar. Another type of glycan structures in which the ^1^H NMR chemical shifts become degenerate are multibranched N-glycans in which the terminal sugar residues are of the same kind, e.g., in *N*-acetyllactosamine-containing disaccharides with terminal β-d-Gal*p* residues capping each branch of the oligosaccharide.^48,49^ From a bisected N-glycan azide, extension of the branches was possible using a galactosyltransferase and UDP-galactose as a donor in a galactosylation reaction followed by reduction of the azido function at the reducing end and derivatization with an ethylenediaminetetraacetic acid (EDTA)-type lanthanide binding tag (Figure 7).

**Figure 8:**
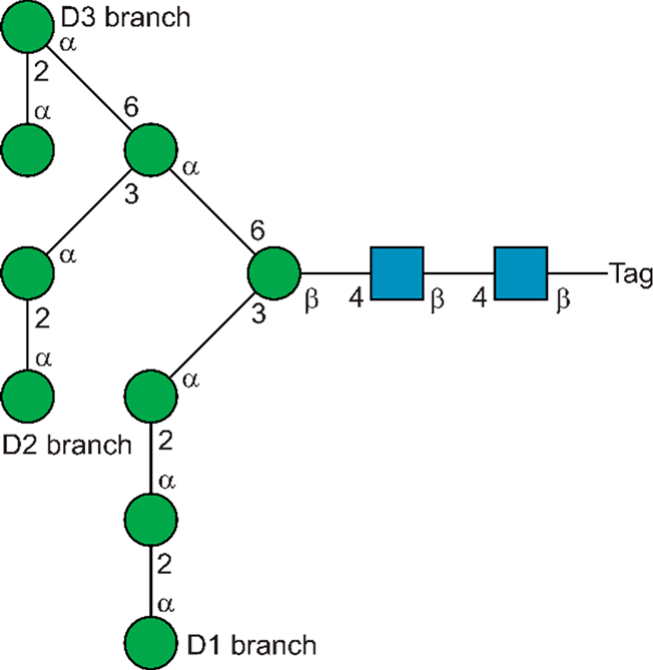
Schematic structure in SNFG format of the high-mannose M9 *N*-glycan containing a Tag with a spin-label or derivatized for lanthanide binding. In the high-mannose M8B N-glycan the terminal α-(1 → 2)-linked mannose residue is absent in the central D2 branch.

Perturbation of the degeneracy of NMR chemical shifts is possible by the introduction of a paramagnetic center in the molecule, which substantially affects the NMR spectrum due to magnetic fields generated by the polarized and rapidly relaxing unpaired electrons of the metal ion.^50^ The paramagnetic effects alter the NMR chemical shifts depending on geometrical relationships between nuclei and the unpaired electron. These effects can be realized by having an ion exhibiting anisotropic magnetic susceptibility attached to the molecule via rigid linker. Due to the *r*^–3^ distance-dependence the pseudocontact shift (PCS) affects nuclei at far greater distances than the dipole–dipole interactions detected by the nuclear Overhauser effect (NOE), which has an *r*^–6^ distance-dependence. Furthermore, an angular dependence is also observed for the PCS related to the principal axis of the magnetic susceptibility tensor. The PCS effects are analyzed as NMR chemical shift differences between an oligosaccharide derivatized to host a paramagnetic ion and the corresponding complex containing a diamagnetic ion as a reference, commonly lanthanum as a La^3+^ ion. Using this strategy, the conformational equilibrium between three main interconverting conformations at the glycosidic linkage of the α-d-Man*p*-(1 → 6)-β-d-Man*p* structural element of a bisected undecasaccharide containing *N*-acetyllactosamine entities could be deduced by analysis of PCS data in which the paramagnetic dysprosium ion Dy^3+^ was complexed with the lanthanide tag.^48^ The conformational states, approximately equally populated, were described as extended with the ω torsion angle in *gauche*-*gauche* and *gauche*-*trans* conformations and a folded conformation with ψ = 90° and ω in the *gauche–gauche* conformation, where the latter conformational state has a significantly higher population compared to a nonbisected biantennary structure in which the β-d-Glc*p*NAc-(1 → 4)-linked residue is absent. Conformational studies on uniformly ^13^C-labeld high-mannose N-glycans in which α-d-Man*p*-(1 → 2)-α-d-Man*p* structural elements are linked to a Man_3_GlcNAc_2_ core via α-(1 → 2)-, α-(1 → 3)- and α-(1 → 6)-linkages (M9 oligosaccharide; Figure 8) or truncated by one residue in which the central D2 branch contains only an α-d-Man*p*-(1 → 3)-linked structure (M8B oligosaccharide) were derivatized with a lanthanide binding tag and utilized thulium as a Tm^3+^ ion complexed with the tag.^51^ NMR PCS data showed significant differences between M8B and M9 for the noncore α-d-Man*p*-(1 → 3)- and α-d-Man*p*-(1 → 6)-linked residues in contrast to those in the α-d-Man*p*-(1 → 2)-α-d-Man*p* structural element α-(1 → 2)-linked to the core. For the conformational analysis the PCS values were back-calculated from replica-exchange MD simulations by which it is possible to explore high-dimensional conformational spaces. The α-(1 → 3)- and α-(1 → 6)-linked branches populate fold-back conformations whereby terminal residues are in spatial proximity with the reducing end residue, though the presence of the terminal mannose in the α-d-Man*p*-(1 → 2)-α-d-Man*p* structural element α-(1 → 3)-linked to the core sterically restricts the fold-back conformations and consequently reduces the conformational flexibility of the larger oligosaccharide M9. Interestingly, by using six different trivalent paramagnetic lanthanide ions and one diamagnetic ion in the study of lactose derivatized with a lanthanide binding tag not only were PCS data possible to obtain, but also RDCs via the alignment introduced by the paramagnetic lanthanide ions.^52^ Importantly, the conformational space sampled included syn, anti-ϕ and anti-ψ states populated to a significant degree thereby underscoring the inherent flexibility at the glycosidic linkage of the disaccharide. The continued development and refinement of rigidified chelators or conformationally locked lanthanide chelating tags, by which different paramagnetic susceptibility tensors can be obtained depending on the lanthanide ion employed,^53^ promise unveiling of conformational states and dynamics that are difficult to assess by other methods. These chelators should, possibly in a modified form, be applicable also to glycans for NMR measurement of PCS.

For conformational studies of glycoproteins with a molecular mass of tens of kilodaltons an alternative approach has been implemented, viz., to insert a lanthanide-ion-binding loop in the protein that can harbor a paramagnetic ion. This was performed for human CEACAM1 containing three N-glycosylation sites where the biosynthesis of the glycans had been restricted to result in M5 oligosaccharides (Man_5_GlcNAc_2_), in which the α-(1 → 6)-linked mannose residue of core is substituted by α-(1 → 3)- and α-(1 → 6)-linked mannose residues, and together with the engineered loop the molecular mass of the construct was ∼18 kDa.^54^ The growth medium was supplemented with, inter alia, d-[1-^13^C]glucose which resulted in isotopic labeling of the anomeric carbon atoms of the glycosyl residues, i.e., d-mannose and *N*-acetyl-d-glucosamine residues, as well as the methyl groups of the latter residues in the oligosaccharide. The PCS displacements in the ^1^H,^13^C-HSQC NMR spectrum using a paramagnetic Terbium(III) ion (Tb^3+^) compared to a diamagnetic Lutetium(III) ion (Lu^3+^) in the loop spanned 5–44 Å for ^13^C-isotopically labeled carbon atoms of isoleucine and alanine residues in the protein, which facilitated optimization of the magnetic susceptibility tensor of the paramagnetic entity, from which spatial regions that will lead to positive or negative PCS displacements can be identified with longer and shorter distances from the paramagnetic ion that would have decreasing and increasing PCS effects, respectively, due to its *r*^–3^ distance-dependence. Conformational clusters were identified from Gaussian accelerated MD simulations and the minimum energy conformation of each cluster was selected for back-calculation of PCS displacements. Furthermore, the proximate *N*-acetyl-d-glucosamine residue of the glycan attached to asparagine 104 was shown to be in close contact with the protein, where the methyl group of its *N*-acetyl group was found to be present in a hydrophobic pocket on the surface of the protein formed by methyl protons of a leucine residue and a ring proton of a histidine residue. Moreover, the interaction and orientation of the amide proton of the *N*-acetyl group was also favored by a hydrogen bond to a side-chain oxygen atom of a serine residue. The other two glycans attached to N111 and N115 were oriented well away from the protein surface. Thus, the structural model that was consistent with PCS data as well as with previously reported NOE data made it possible via MD simulations to identify atomic interactions between one of the glycans and surface residues of the protein.

The NMR spin relaxation of dipole–dipole interactions between an unpaired electron and nuclei in the molecule may also be used to investigate conformation and dynamics via its *r*^–6^ distance-dependence and is referred to as paramagnetic relaxation enhancement (PRE). Conformational preferences of the M9 oligosaccharide (Figure 8) derivatized with a TEMPO-based aminoxyl radical was investigated by PRE methodology.^55^ The difference in transverse relaxation rates *R*_2_ of the anomeric protons in the α-linked mannosyl residues were monitored prior to and after radical quenching by ascorbic acid corresponding to the paramagnetic and diamagnetic states, respectively. The α-(1 → 6)-linked residue being part of the Man_3_GlcNAc_2_ core was affected the most; i.e., the difference in *R*_2_ values for its anomeric proton was the largest. The terminal residues in the D2- and D3-branches were influenced second to the α-(1 → 6)-linked residue of the core, followed by the penultimate residues in these branches and the α-(1 → 3)-linked residue of the core. Least impact caused by the spin-label was observed for the α-d-Man*p*-(1 → 2)-α-d-Man*p* structural element of the D1-branch. Taken together these results indicate a fold-back of the D2- and D3-branches, consistent with the PCS data obtained by the paramagnetic lanthanide ion derivative of the M9 oligosaccharide highlighting the complementarity of the PCS and PRE techniques once a spin-label has been introduced as tag in the molecule.

**Figure 9:**
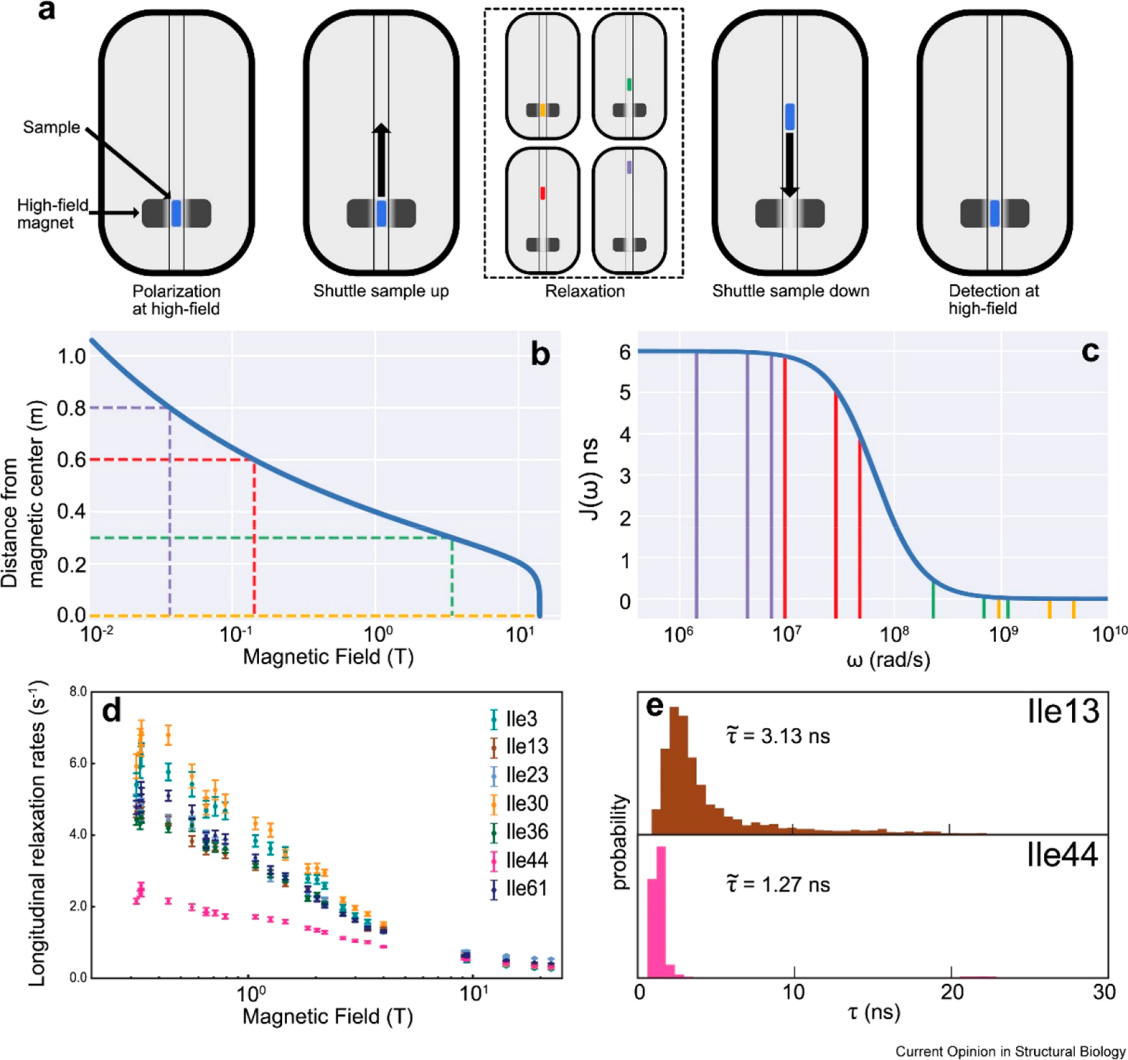
Principle of high-resolution relaxometry. (a) After polarization at a high magnetic field, the sample is transferred to a specific position in the stray field for relaxation. This is followed by shuttling down into the high field for detection. Four experiments with different transfer distances are represented by the colors yellow, green, red, and purple with traveling distance of 0, 0.3, 0.6, and 0.8 m, respectively. (b) Magnetic field profile as a function of the distance from the magnetic center with the four distances from the magnetic center. (c) Spectral density of a carbon-13 nucleus, coupled to a single proton in a protein with a correlation time of 15 ns with the frequencies sampled by longitudinal relaxation of the carbon-13 nucleus at the four magnetic fields. (d) Magnetic-field dependent longitudinal relaxation rates of the ^13^C nucleus in specifically labeled ^13^C^1^H^2^H_2_ δ1 methyl groups of isoleucine residues in the protein ubiquitin. (e) Probability distribution of the correlation time for ns motions in residues isoleucine 13 and 44 in ubiquitin. Reproduced with permission from ref (58). Copyright 2022 Elsevier.

Investigation of conformational dynamics of oligo- and polysaccharides on the picosecond to nanosecond time scale can be performed by measurement of different ^13^C nuclear spin relaxation rates at several magnetic field strengths, which was performed for a site-specifically ^13^C-isotope labeled disaccharide employing seven magnetic fields ranging 7–22 T, corresponding to ^1^H frequencies of 300–950 MHz.^56^ However, even though several magnetic fields were used in the study the range is still limited. An alternative for quantification of motions on nanosecond time scales close to the correlation time for overall rotational diffusion is high resolution relaxometry,^57^ especially since measurement of relaxation rates can be performed over more than 2 orders of magnitude, from ∼0.1 T to the currently highest magnetic field at ∼28 T. The technique relies on polarization of the sample at high field whereafter it is shuttled to a stray field by displacing it upward to a specific position for relaxation, followed by transferring it downward into the high field for detection (Figure 9).^58^ This ensures high sensitivity and high resolution as polarization and detection take place at high field. Magnetic field profiles, spectral density profiles and magnetic-field dependence of relaxation rates can thus be monitored followed by interpretation of data to unravel molecular motions and dynamics, hitherto performed for proteins. We envisage that continued developments of the high resolution relaxometry methodology should facilitate insight into the dynamics of structurally complex glycans.

Hydrogen bonding in carbohydrates in water or water:dimethyl sulfoxide mixtures is elusive but transient inter-residue hydrogen bonding like that depicted for β-panose (Scheme 1) has been supported by NMR experiments determining hydroxyl proton exchange rates or detecting magnetization transfer between sugar residues involving a hydroxyl proton,^59,60^ thereby confirming the presence of a classical hydrogen bond. The IUPAC recommendations of the definition of the hydrogen bond is given by “The hydrogen bond is an attractive interaction between a hydrogen atom from a molecule or a molecular fragment X–H in which X is more electronegative than H, and an atom or a group of atoms in the same or a different molecule, in which there is evidence of bond formation”, together with six criteria, inter alia, “The X–H**···**Y–Z hydrogen bond leads to characteristic NMR signatures that typically include pronounced proton deshielding for H in X–H, through hydrogen bond spin–spin couplings between X and Y, and nuclear Overhauser enhancements.”^61^ Furthermore, hydrogen bonding is generally described to be due to polarization and charge transfer interactions, with a significant contribution from ionic valence bond structures.^62^

**Figure 10:**
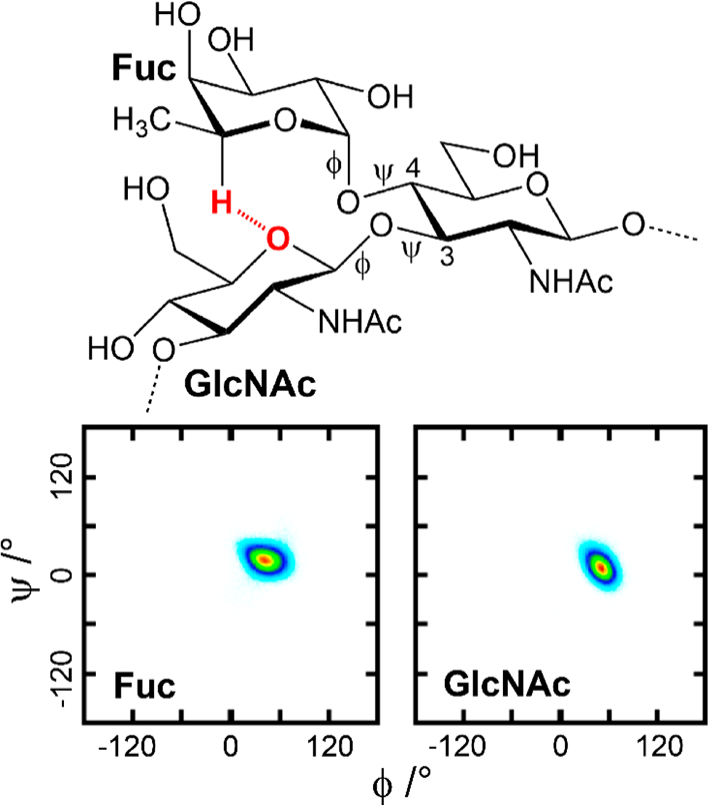
(Top) Schematic representation of sugar residues in the branching region of the repeating unit of the *E. coli* O159 O-antigen polysaccharide, depicting a possible nonconventional hydrogen bond between H5 in l-fucose and O5 in *N*-acetyl-d-glucosamine. (Bottom) Two-dimensional distributions from MD simulations of *E. coli* O159 LPS^68^ having glycosidic torsion angles ϕ and ψ in the syn-state with an exoanomeric conformation for α-l-Fuc*p*-(1 → 4)-d-Glc*p*NAc (left) and β-d-Glc*p*NAc-(1 → 3)-d-Glc*p*NAc (right). The population distributions were selected based on the interatomic distance between H5@Fuc (side-chain) and O5@GlcNAc (nonbranched residue) being less than 2.7 Å (i.e., the sum of their van der Waals radii) and correspond to ∼40% of the MD simulation. The density was rescaled by the maximum value: white for 0, blue for 0.1, green for 0.3, yellow for 0.7, and red for 1. Adapted from ref (68). Copyright 2020 American Chemical Society.

In a description of carbohydrates, either primary^63^ or three-dimensional^64^ structures have been relied on, in contrast to proteins for which also secondary structure elements are part of the classification, where the local structure of the protein backbone is stabilized by intramolecular hydrogen bonds between amide groups resulting in the commonly observed types α-helix and β-strand. The uniqueness of carbohydrate structures is that they can be branched. In fucosylated glycopeptides, secondary structural elements were identified with consensus motifs of the kind β-d-Gal*p*-(1 → 4)[α-l-Fuc*p*-(1 → 3)]-d-Glc*p*NAc (Lewis^x^) and α-l-Fuc*p*-(1 → 4)[β-d-Gal*p*-(1 → 3)]-d-Glc*p*NAc (Lewis^a^), in which nonconventional hydrogen bonding was proposed to occur between H5 in fucose and O5 in galactose residues,^65,66^ a finding that could be confirmed by long-range ^1^H,^13^C-HSQC NMR experiments correlating H1 in galactose and C5 in fucose in a sialyl-Lewis^x^ pentasaccharide.^67^ This type of geometrical arrangement is also present in polysaccharides such as the *E. coli* O159 O-antigen (Figure 10), and MD simulations of the LPS show that the interatomic distance between H5@Fuc (side-chain) and O5@GlcNAc (nonbranched residue) being less than the sum of their van der Waals radii (2.7 Å) corresponds to ∼40% of the MD simulation, suggesting that also in this case a nonconventional hydrogen bond may be present.^68^ Nonconventional hydrogen bonding has also been proposed in linear oligosaccharides containing the structural element α-d-Gal*p*-(1 → 4)-α-d-Gal*p* in which the inter-residue C5′-H5′···O3 interaction makes a favorable contribution of ∼2 kcal·mol^–1^ based on natural bond orbital calculations.^69^ By NMR experiments, the hydrogen bonded conformation of Lewis antigens has been shown to be favored by ∼1 kcal·mol^–1^ over the one lacking the nonconventional hydrogen bond for the two conformations in rapid exchange.^70^ By combining natural carbohydrate motifs with β-(1 → 4)-linked cellulose oligomers as strands with a turn unit in the form of a Lewis-type trisaccharide structure based on nonconventional hydrogen bonding in conjunction with classical hydrogen bonding and hydrophobic interactions between strands it was possible to form glycan hairpin structures having a folded conformation.^71^ In the identification of 3D epitopes important for recognition of glycans in naturally occurring structures, construction of foldamers that adopt well-defined conformations in solution and for scaffolds based on carbohydrates, the secondary structure elements that they are based upon should be analyzed and discerned. Future work to establish a general classification of secondary structure elements for glycans, like for proteins, will not only be interesting and challenging but should reveal fundamental aspects of glycan structure and how it affects recognition processes in different environments.

## Interactions (Recognition)

Glycan–protein interactions are determined by, inter alia, CH−π interactions,^72,73^ stereoelectronics,^74^ and hydrogen-bonding networks.^75^ These interactions are usually investigated by more than one experimental technique. An oligosaccharide having a short reorientational correlation time shows in general positive NOEs or absence of NOEs in ^1^H,^1^H-NOESY NMR experiments. However, if it binds, in rapid exchange, to a protein having a long global correlation time the change in sign of the NOE for the oligosaccharide to negative facilitates determination of the conformation when bound to the protein. This transferred NOESY (tr-NOESY) technique^76^ was used in combination with other NMR experiments to investigate the conformation of a Lewis^x^ trisaccharide (*M*_w_ 529 Da) when bound to the carbohydrate binding domain (CRD) of the C-type lectin DC-SIGN compared to its conformation in solution.^77^ To make the tr-NOESY approach more efficient a slowly tumbling tetrameric form of DC-SIGN was used with a molecular mass of 160 kDa. Interestingly, based on intramolecular NMR-derived proton–proton distances the conformation of the trisaccharide bound to the lectin differed from that reported in solution without protein. Notably, it also differed to that of the crystal structure complex of the CRD of DC-SIGN, highlighting differences in structure and the dependence on environment. The tr-NOESY technique has been extended to studies of intermolecular interactions in small- and medium-sized protein complexes by using ^13^C-edited/^13^C-filtered NMR experiments and is applicable to weakly bound complexes as long as the exchange is fast relative to the inverse of the ^1^H *T*_1_ relaxation times of the constituent components.^78^

The NOE across the glycosidic linkage between sugar residues often shows only one proton–proton interaction that is strong (correlation time of the molecule permitting) and evident, though detailed analysis may reveal additional NOEs that will be important for a conformational analysis of the molecule. Sufficient information may still be lacking to define the bound conformation of an oligosaccharide-protein complex by tr-NOESY NMR experiments. In 6-deoxyhexoses, e.g., fucose and rhamnose, the exocyclic group is a methyl group and the ^1^H NMR chemical shifts are observed at ∼1 ppm, i.e., far away from the bulk region of those from carbohydrates, thereby facilitating additional NOE interactions to be identified. However, the correlation times of the rapidly spinning methyl groups are short and any interactions between methyl and methine groups will have a different effective correlation time than those between pairs of methine groups in the carbohydrate–protein complex. Thus, by utilizing also the methyl-methine NOEs, when available, enough information may be gained, in conjunction with MD simulations, to deduce the conformation of an oligosaccharide in complex with a protein. This extension of the methodological repertoire using effective proton–proton distances within a bound octasaccharide, which contains rhamnosyl residues, in complex with the *Shigella flexneri* bacteriophage Sf6 tailspike protein together with two-dimensional ϕ,ψ distance-plots from MD simulations made it possible to define the bound conformation of the octasaccharide in the complex, in excellent agreement with the crystal structure of the complex.^79^

**Scheme 2:**
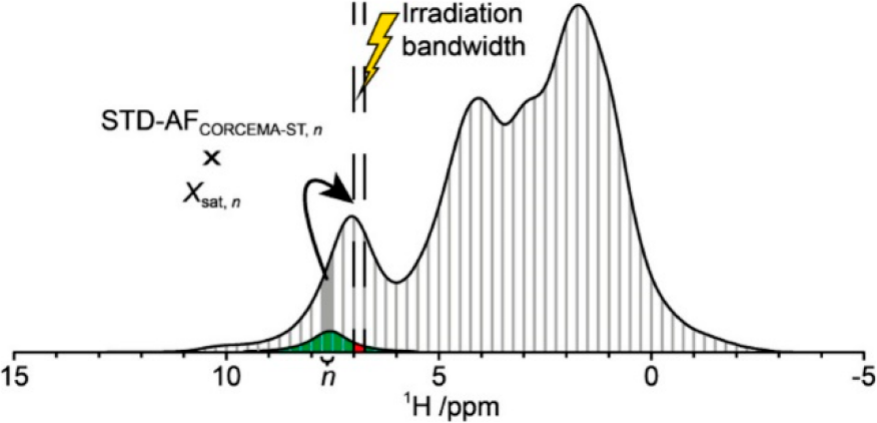
Concept of the CORCEMA-ST-CSD Approach The spectrum is divided into separate regions (*n*) and for each one a CORCEMA-ST simulation is performed to generate a set of STD-AF_CORCEMA-ST,*n*_. Each region is then multiplied with X_sat,*n*_, i.e., the part of the chemical shift distribution for the specific region that is within the irradiation bandwidth (the red area) divided with full distribution (the sum of the red and green areas). The final STD-AF_CSD_ is calculated as the sum of all STD-AF_CORCEMA-ST,*n*_ in the spectrum. Reproduced from ref (82). Copyright 2016 American Chemical Society.

Subsequent to elucidation of the conformation of a bound ligand by tr-NOESY NMR experiments the binding epitope of the ligand recognized by a protein can be investigated by saturation transfer difference (STD) NMR experiments.^80^ In the technique, proton resonances of a protein, different from those of the ligand, are saturated and in transiently formed complexes this saturation affects the bound ligand whereby its proton peak intensities are attenuated in relation to proximity of the ligand to the protein. A difference spectrum obtained from off- and on-resonance NMR spectra will reveal ^1^H NMR chemical shifts of ligand protons in close contact with the protein, in processes with *K*_d_ ∼10^–3^–10^–7^ M. Comparison of protein:ligand complexes determined by X-ray crystallography with results from STD NMR experiments can give insight into if crystal structures are representative of recognition events in solution,^80^ which for the hexasaccharide portion of the ganglioside GD1a, i.e., the oligosaccharide devoid of the ceramide moiety, bound to botulinum neurotoxin A was investigated employing full relaxation-matrix calculations of STD amplification factors (STD-AF) where the ligand/protein ratio used in the experiments is taken into account.^81^ The buildup curves, as a function of saturation time, resulted in excellent agreement between those based on the crystal structure complex and the ones obtained from the solution-state STD NMR experiments, indicating a high degree of predisposition in the recognition events. However, for proteins the resonances can be broader than the narrow bandwidth of the saturation pulses and only resonances with chemical shifts within a defined irradiation frequency would be fully saturated, whereas those close to the irradiation interval would be only partially saturated. To alleviate this limitation in the description of “to what extent resonances are saturated,” the chemical shift dispersion (CSD) of protein resonances were considered whereby the complete relaxation and conformational exchange matrix theory adapted for the saturation transfer measurements (CORCEMA-ST) was extended to CORCEMA-ST-CSD (Scheme 2).^82^ By looping the CORCEMA-ST calculations in narrow intervals over the whole spectral region the contributions to spectral intensity within a defined irradiation bandwidth were calculated and summed over all intervals, resulting in overall better agreement between simulated and experimental data.

**Figure 11:**
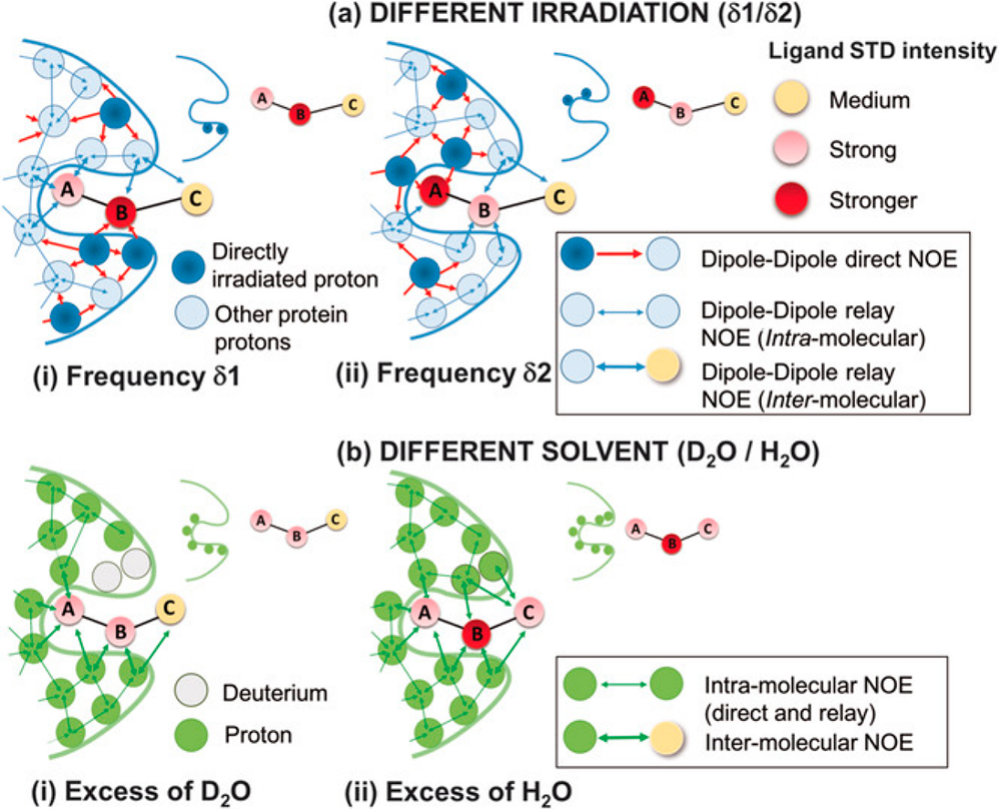
Cartoon representing the two implementations of the DEEP-STD NMR method. (a) Different irradiation frequencies: ligand protons receive larger saturation if the protein protons in their proximity are “directly irradiated” instead of “relayed-NOE” saturated. STD NMR is carried out with selective irradiation (δ1) on protein protons close to ligand proton B (i) and with selective irradiation (δ2) on protein protons close to ligand proton A (ii). The distinct binding epitopes of the ligand are sketched in the free state. (b) Different solvent composition: ligand protons close to slowly exchanging protein exchangeable protons receive less saturation if the latter are exchanged to deuterium (in D_2_O) instead of a proton (in H_2_O). STD NMR experiments are thus carried out in D_2_O (i) and H_2_O (ii). Reproduced from ref (83). Copyright 2017 Wiley-VCH.

To obtain additional information on the binding epitope of the ligand and amino acids of the protein receptor involved in the recognition process, a methodology was devised relying on differential epitope mapping by STD NMR (DEEP-STD NMR) (Figure 11).^83^ The approach relies on running pairs of STD NMR experiments where the protein protons directly irradiated will affect the protons of the ligand more than the protein protons that relay magnetization from the directly irradiated protons. By performing irradiations of, on the one hand, aromatic protons and, on the other, aliphatic protons of the protein, differences can be identified and DEEP-STD factors can be calculated thereby highlighting prominent interactions. A second variant of the methodology is to investigate contributions related to polar amino acids in the binding pocket that have side-chains with exchangeable protons. In water these protons can contribute to additional saturation transfer in comparison to deuterium oxide and slowly exchanging protons are expected to make the largest differences in STD effects. Likewise, DEEP-STD factors can be computed to give information on proximity between ligand and amino acids in the binding pocket.

**Figure 12:**
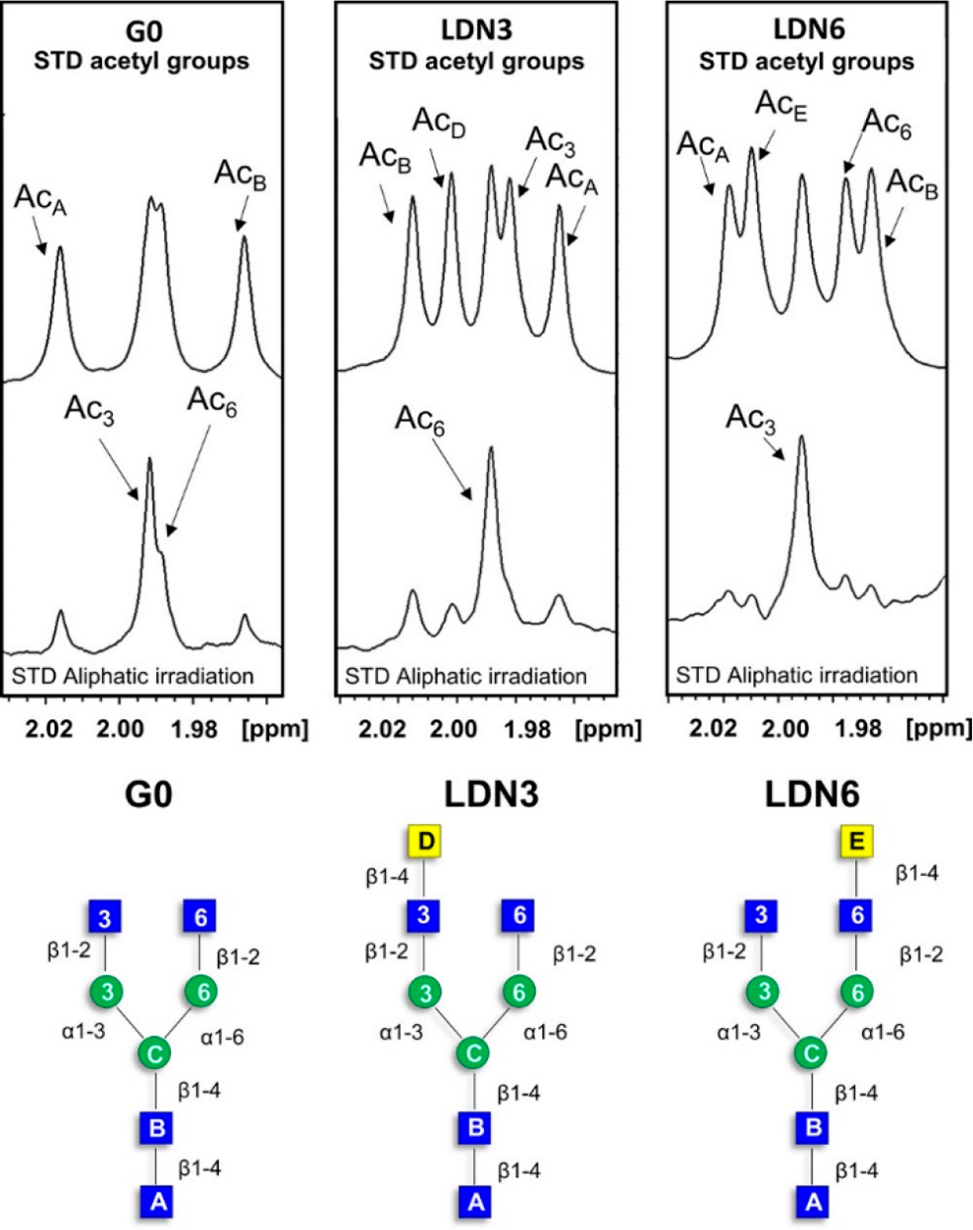
Acetyl region of the GlcNAc residues in the STD NMR spectra (bottom). Corresponding off-resonance NMR spectra (top). (below) From left to right: the complexes of LSECtin with G0, LDN3, and LDN6, respectively. Reproduced from ref (84). Copyright 2022 American Chemical Society.

The importance of investigating interactions by more than a single biophysical method is highlighted by the interaction studies of the C-type lectin LSECtin that recognizes as a minimum epitope the structural element β-d-Glc*p*NAc-(1 → 2)-d-Man*p* (GN2M) in biantennary *N*-glycans.^84^ STD NMR experiments revealed that when GN2M is available for binding as a terminal nonsubstituted entity it is better recognized when it is α-(1 → 6)-linked than when it is α-(1 → 3)-linked (Figure 12). Furthermore, and importantly, when one of the branches is capped by a terminal d-Gal*p*NAc residue, recognition of the GN2M moiety is equally accessible, in stark contrast to glycan array studies where only the α-(1 → 6)-linked GN2M entity was efficiently recognized by the lectin. Further developments of STD methodologies include the universal saturation transfer analysis (uSTA) method that enables quantitative determination of precise binding rates and dissociation constants whereby the severe acute respiratory syndrome coronavirus 2 (SARS-CoV-2) spike trimer was shown to bind sialic acid-containing oligosaccharides in an end-on manner.^85^ However, whereas the dissociation constants (*K*_D_) were on the order of 10^–5^ M in the study by Buchanan et al.,^85^ those reported by Maass et. al^86^ showed weaker binding with *K*_D_ values on the order of 10^–2^ M for the sialoglycan trisaccharides interacting with the spike protein. The large differences in the dissociation constants will require further analysis to resolve the origin of their lack of agreement. Other STD-based studies utilized an interligand STD (IL-STD) NMR method applied to cholera toxin subunit B and two inhibitors adjacently bound within the GM1 (a ganglioside with a pentasaccharide structure containing a sialic acid side-chain) binding site,^87^ and an imaging STD NMR methodology in which chemical shift imaging and controlled concentration gradients of small molecule ligands are combined with STD experiments to determine protein–ligand dissociation constants, inter alia, of *N*-acetyl-d-glucosamine binding to wheat germ agglutinin.^88^

The complexity of saturation magnetization transfer (SMT) experiments may lead to artifacts, and some of them have been identified originating from the use of long saturation pulses resulting in changes in the signals of proximate peaks, oversaturation of the NOE information due to strong saturating radio frequency fields, and the performance of ^15^N decoupling during the course of the experiments on ^15^N-labeled samples; remedies to alleviate these artifacts were proposed leading to higher quality data from SMT experiments.^89^ A number of different variants of the original STD NMR experiment have been developed to date, which facilitates the selection of suitable experimental conditions for future applications of the technique in studies of binding processes between carbohydrates and proteins. Furthermore, complementary to STD NMR studies is the recording of ^1^H,^15^N-HSQC NMR spectra of proteins in the absence and presence of ligands from which 2D cross-peak chemical shift perturbation (CSP) data can be obtained revealing the binding region and the active site of the protein that recognizes glycans or binds to inhibitors.^77,90^

Isotopic labeling of proteins by ^15^N, ^13^C, and/or ^2^H facilitates studies of protein structure and dynamics but also interaction studies with ligands. Uniform ^13^C,^15^N-labeling of N-glycans can be introduced by a biosynthetic methodology using carcinoma cells infected by an adenovirus construct encoding for HIV-1 gp120, in which half of the glycoprotein mass is composed of high-mannose-type glycans thereby providing good amounts of labeled glycans.^91^ To study the conformation of the M9 oligosaccharide (cf. structure in Figure 8) when bound to the lectin microvirin, CSPs in ^1^H,^15^N-HSQC NMR spectra were monitored using M9 at natural abundance and ^15^N-labeled protein. Notably, every perturbed 2D cross-peak was shifted to two distinct chemical shifts and it was concluded that interactions between microvirin and an epitope at the D1-branch were more extensive than those with the D2- or D3-branches. The uniformly ^13^C-labeled M9 enabled NMR studies of the conformation of the ligand in complex with the lectin based on, inter alia, ^13^C-edited NOESY experiments which revealed a quite similar conformational space as unbound M9, though binding of microvirin to the D1-branch takes place via two slightly different epitopes. In contrast, the interactions between the lectin DC-SIGN and ^13^C-labeled M9 studied by ^1^H,^13^C-HSQC spectroscopy revealed that upon formation of a 1:1 complex between ligand and protein, signals from sugar residues at the core region common to all N-linked oligomannose structures, i.e., in this case α-d-Man*p*-(1 → 6)-β-d-Man*p*-(1 → 4)-β-d-Glc*p*NAc-(1 → 4)-d-Glc*p*NAc, disappeared thereby indicating the binding region and that the binding process takes place on a slower time scale of ∼10 ms.^91^ Chemical synthesis using ^13^C-uniformly labeled monosaccharides is an alternative to obtain well-defined oligosaccharides and this approach was used to make α-d-Man*p*-(1 → 2)-α-d-Man*p*-(1 → 6)-α-d-Man*p*-OMe (M26).^75^ The complex between ^15^N-labeled cyanovirin-N (CV–N) and M26 was studied by ^1^H,^13^C-CT-HSQC, ^1^H,^13^C-HSQC-NOESY and ^1^H,^13^C-HSQC-TOCSY NMR experiments with focus on hydroxyl proton torsion angles of the ligand in the bound conformation, which revealed that specific rotamers of HO3 and HO4 hydroxyl groups in the terminal and central sugar residues were preferred in the CV–N:M26 complex. Enzymatic synthesis was employed for poly-*N*-acetyllactosamines, which have a repetitive structure of →3)-α-d-Gal*p*-(1 → 4)-β-d-Glc*p*NAc-(1→ disaccharide entities, with up to hexasaccharides having ^13^C-uniformly labeled galactose residues at different positions in the sequence of alternating sugar residues and was facilitated by β1,3-*N*-acetylglucosaminyltransferase in the presence of UDP-GlcNAc and β1,4-galactosyltransferase in conjunction with UDP-UL-^13^C_6_-galactose.^92^ The binding preferences of various galectins from different families were then investigated by 2D STD-^1^H,^13^C-HSQC NMR experiments to carry out epitope mapping and to discriminate between terminal, internal and nonterminal epitopes of the *N*-acetyllactosamine-containing oligosaccharides. The STD-^1^H,^13^C-HSQC NMR experiment was also utilized for interaction studies of the SARS CoV2 spike glycoprotein in conjunction with sialic acid-containing trisaccharides, uniformly ^13^C-labeled at the sialic acid and galactose residues.^93^

The broad dispersion of ^19^F NMR chemical shifts and hydroxyl-to-fluorine substitution of monosaccharides makes it possible to map the importance of hydroxyl groups in carbohydrate–protein recognition processes.^94^ Interaction studies with proteins were carried out using ^19^F{^1^H} CPMG *T*_2_-filtered NMR experiments with a short and a long relaxation time, analyzed as difference spectra as well as by addition of known ligands to the proteins in competition experiments. Using a library of fluorinated monosaccharides, it was possible to (i) define sugar selectivity, (ii) detect anomeric preference, and (iii) identify hydroxyl groups important for binding. An alternative approach using ^19^F as a reporter group to probe binding for Lewis type 2 glycans was to make ^19^F-containing analogues with the labeling at position 3 of glucose in the inner core lactose subunit, a position far from the anticipated structural part of the oligosaccharide forming the binding-epitope.^95^^19^F{^1^H} CPMG *T*_2_-filtered NMR experiments were also used to detect weak binders to lectins and ^19^F NMR was used for real-time enzyme kinetics, either by monitoring a β-galactosidase hydrolyzing a ^19^F-labeled lactose or a sialic acid transferase adding Neu5Ac to the galactose residue of the ^19^F-derivatized lactose. A complementary approach based on the recently developed 2D ^1^H,^19^F STD-TOCSYreF NMR experiment^96^ was used to study the interaction between DC-SIGN and the site-selectively ^19^F-labeled mannosyl-containing portion of the M9 oligosaccharide (cf. Figure 8 for the M9 structure) revealing the importance of the D2-branch in the interaction with its receptor.^97^ A different approach to utilize ^19^F in NMR studies of carbohydrate–protein interactions is to incorporate fluorine in amino acids of the protein. Tryptophan is often present in the binding pocket of carbohydrate-binding proteins,^73^ and 5-fluoroindole was used as a precursor of 5-fluoro-tryptophan labeling of LecA for protein-observed ^19^F NMR studies.^98^ The lectin contains four tryptophan residues and NMR assignment of their resonances was obtained by selective mutation to phenylalanine residues. Upon addition of Ca^2+^ and galactose to the apo-form of the lectin, CSPs were observed. Importantly, the method was shown to be sensitive for identification of weak binders. Stable 5-fluoro-tryptophan labeling of tryptophan residues was also utilized to study the glycosyl transferase WaaG from *E*. *coli* that transfers a glucosyl residue to a heptose which is part of the inner core region of its LPS. Importantly, a ^19^F NMR chemical shift change upon binding UDP-glucose indicates that the environment is changed around a tryptophan residue even though it is not directly involved in the interaction at the donor binding site, which indicates that a larger structural movement such as interdomain rearrangements takes place.^99^ Like ^19^F the isotope ^77^Se is an NMR-active spin-1/2 nucleus; its natural abundance is 7.6% with a large spectral range of ∼3000 ppm. By synthesizing methyl 1-seleno glycosides, binding to and selectivity of lectins were possible to detect by ^77^Se{^1^H} NMR.^100^ The advantages are straightforward detection without any interfering background signals and that the modification as a selenoglycoside takes place at the reducing end which should not affect the binding epitope of the ligand. However, significantly higher sensitivity can be obtained by using ^1^H,^77^Se out-and-back NMR experiments.^101^ A selective heteronuclear ^1^H↔^77^Se Hartmann–Hahn transfer (HeHaHa) NMR experiment was developed to this end and used in interaction studies of a selenodigalactoside and human lectins. Furthermore, ^77^Se-enrichment (99%) can be obtained from elemental selenium and used for chemical synthesis of ^77^Se-containing oligosaccharides.^102^ Isotopic labeling of either glycan or protein or both^103^ enables detailed investigations of carbohydrate–protein interactions as exemplified by the above and will for antibodies, which contain N-glycans linked to the Fc portion of the molecule,^12^ and glycoproteins in general be of great importance by facilitating studies that can progress our understanding of biomolecular processes.

**Figure 13:**
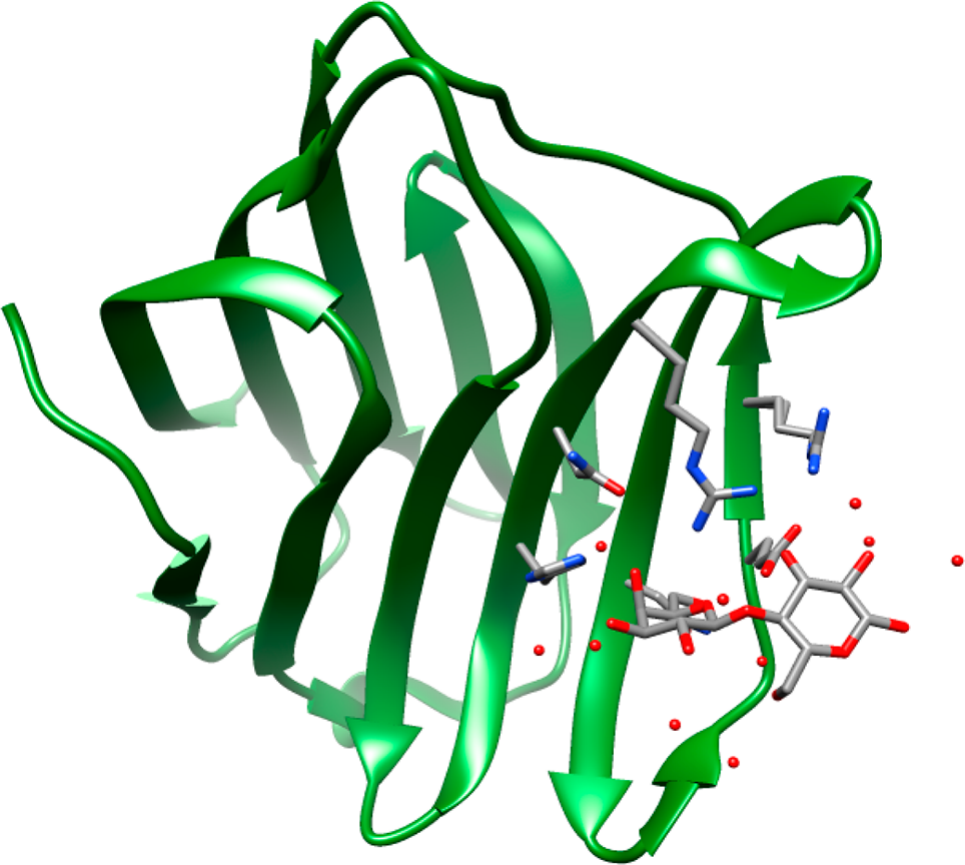
Crystal structure of galectin-3 with lactose (represented as the β-anomeric form at its reducing end) in the binding site of the protein (PDB entry 3ZSJ).^104^ The protein structure is shown by a ribbon diagram (green) and side-chains of selected amino acids close to the ligand are drawn explicitly. Atoms are colored gray (carbon), blue (nitrogen) and red (oxygen). Water molecules that coordinate the disaccharide or mediate hydrogen bonds between lactose and the protein are shown by red spheres. The molecular model was made using UCSF Chimera.^141^

A striking aspect of lectins and carbohydrate-binding modules of enzymes is the complementarity of the protein and the carbohydrate ligand to be recognized. This is exemplified by the ultrahigh-resolution crystal structure of the carbohydrate recognition domain of galectin-3 (Gal3C) in complex with lactose and in its apo form.^104^ The high resolution of 0.86 Å obtained for the complex is similar to that of small-molecule X-ray crystallography, e.g., 0.60 Å for a disaccharide,^105^ and key water molecules in the apo form occupy the same positions as oxygen atoms of lactose in the complex, revealing that the binding site of Gal3C is preorganized to recognize terminal β-d-galactosides. In the complex there are water molecules that mediate hydrogen bonding between lactose and Gal3C as well as those that coordinate the disaccharide but not the protein (Figure 13). Residence times of water molecules bound to Gal3C were investigated using ^2^H NMR longitudinal relaxation dispersion experiments covering almost two orders of magnitude in the frequency range 2–92 MHz. The profile of the relaxation dispersion experiments for apo-Gal3C and Gal3C in complex with lactose was closely similar, which indicates that the disaccharide does not displace long-lived water molecules from the binding site; consequently, the water molecules exchange with bulk water on a time scale of nanoseconds or faster at room temperature.

## Outlook and Summary

Protein structures determined by cryo-EM will aid and facilitate NMR studies of molecular systems where lectins recognize carbohydrates, enzymes hydrolyze glycans or glycosyl transferase mechanism and specificity are investigated. The continued development of NMR pulse sequences, whether to improve resolution in NOE-based spectra,^106^ measure ^1^*J*_CH_ accurately for application in RDC-based studies^107^ or ^n^*J*_HF_ in complex NMR spectra,^108^ determine NMR spin relaxation parameters by accordion spectroscopy,^109^ or acquire 2D NMR spectra more efficiently using NOAH experiments,^110^ will impact the use liquid-state NMR spectroscopy for analysis of glycan conformation and dynamics. Due to the limited spectral dispersion of, in particular, ^1^H NMR resonances of glycans and glycoconjugates, the increase of the highest magnetic fields available as a result of improved technology of high-temperature superconductors^111^ has resulted in magnets operating at 1 GHz ^1^H frequency^112^ and higher, thereby improving resolution in NMR spectra. Improved sensitivity resulting from cryogenically cooled NMR probes leading to a gain by one order of magnitude in time spent acquiring NMR data, compared to room temperature probes, is now well-established. A leap in sensitivity has been shown feasible by dynamic nuclear polarization (DNP) NMR spectroscopy, which may result in sensitivity gains of up to four orders of magnitude,^113^ using hyperpolarized water^114^ for solutions containing, inter alia, monosaccharides.^115^ Using this NMR methodology to identify small molecules from fragment-based libraries as scaffolds should be possible to perform efficiently when exploring donor and acceptor binding sites in glycosyltransferases in order to inhibit their activities, as a basis for drug developments. To study structural requirements and interactions between glycans and proteins as well as proteins assemblies that are required in order to be functional, bicelles or nanodiscs may become suitable systems to this end,^116,117^ utilizing, among other things, ^13^C-labeling of methyl groups for liquid-state NMR studies as the size of the multicomponent systems is significantly larger than of the glycans or proteins per se.

**Figure 14:**
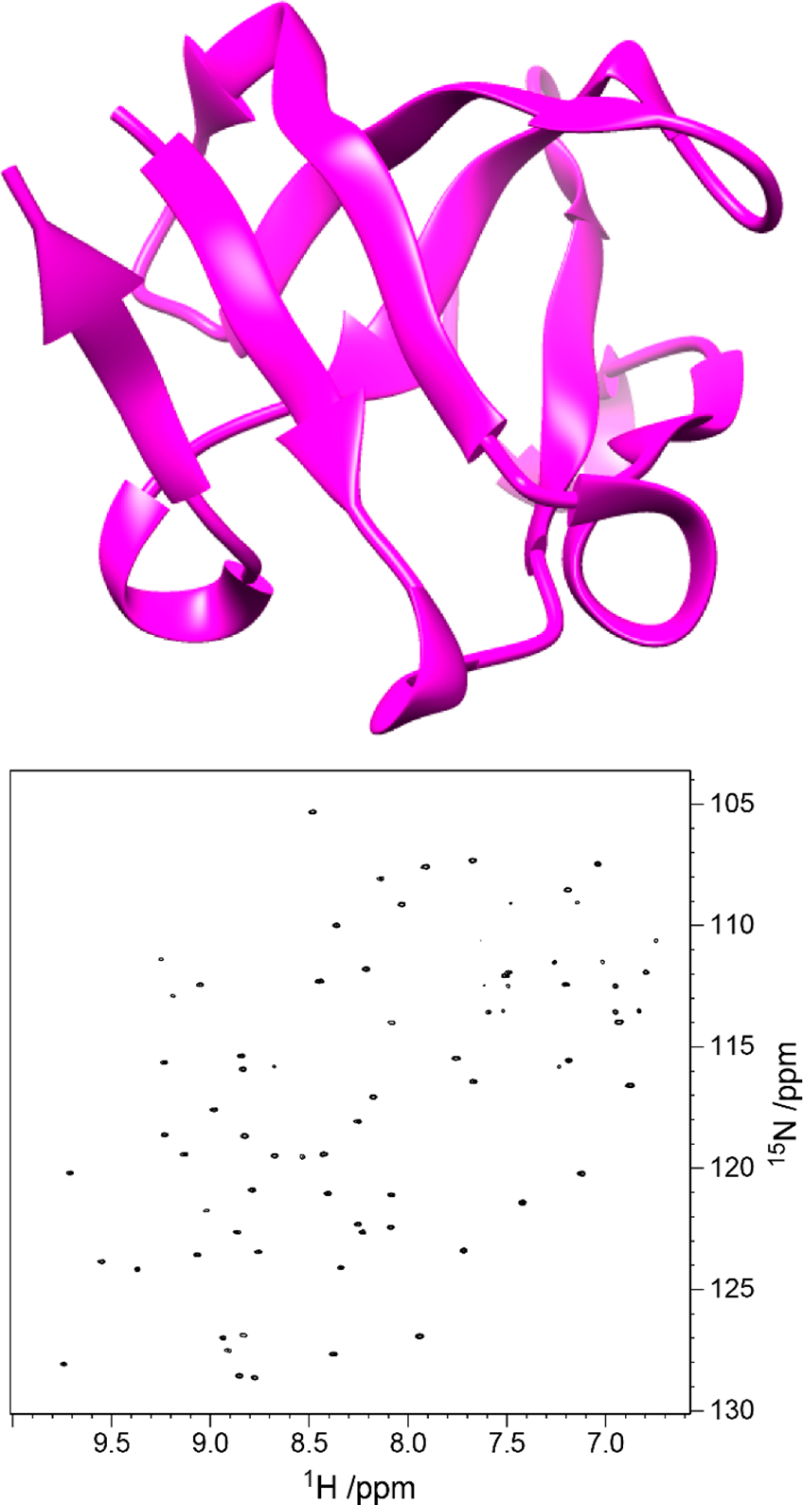
(Top) Three-dimensional structural model of the C-terminal domain of an amidase generated by AlphaFold 2.^123^ The molecular model was made using UCSF Chimera.^141^ (Bottom) ^1^H,^15^N-HSQC NMR spectrum at natural abundance (298 K, 96% H_2_O/4% D_2_O) of a construct of the C-terminal domain. The well-dispersed resonances in the 2D NMR spectrum indicate that the protein is structured, as indicated by the AlphaFold model (E. R. Scaletti, P. Stenmark, and G. Widmalm, unpublished results).

Artificial intelligence including machine learning and deep learning, where the latter uses artificial neural networks and is a subset of machine learning, are methods revolutionizing science. The ability to predict NMR chemical shifts of molecules by machine learning,^118−120^ understand the biology of branched *N*-glycans,^121^ or unveil the origin of glycan-mediated host-microbe interactions^122^ by deep learning methods opens new avenues for exploring the importance of glycan structure in biology using NMR spectroscopy as the experimental technique. With the introduction of AlphaFold^123^ to predict three-dimensional structure of proteins, analysis of carbohydrate binding proteins, structures of glycosyl transferases,^124^ and possible functions of proteins as well as modules thereof has been completely changed. One can now with confidence first investigate whether a sequence of amino acids of a protein or a subset of these have a defined three-dimensional structure, and if this is the case, a construct can be made to check the prediction by NMR spectroscopy (Figure 14), prior to trying to elucidate the function of the module or of the whole protein. Investigation of the catalytic domain of the rat α-2,6-sialyltransferase ST6Gal I was previously based on PRE NMR experiments using TEMPO spin-labeled analogues of both the donor CMP-Neu5Ac and the disaccharide acceptor LacNAc to elucidate which amino acids constitute the binding site and to determine the orientation of the acceptor molecule in the binding site,^125,126^ but without access to a 3D structure of a mammalian ST6Gal I, which has low sequence similarity to bacterial sialyltransferases, for which there were crystal structures at the time. Subsequent to these NMR studies, the crystal structures of both rat and human ST6Gal I were determined,^127,128^ which facilitate further analysis of the recognition process between glycosyl transferase, donor and acceptor molecules. PRE NMR experiments can thus be used to obtain important information about recognition processes in solution, and with the advent of AlphaFold future PRE-based studies would have access to a structural model for the interpretation of experimental NMR data. Unraveling and delineating dynamic communication networks of key allosteric interactions can be aided by NMR experiments^129,130^ and cooperatively controlled processes in which each binding step is under control of a previous binding event have been described,^131^ as well as allosteric regulation of lysosomal enzyme recognition and druggable allosteric sites in lectins.^132,133^ The subtle interaction processes, which may be unveiled to some extent by NMR spectroscopy experiments, should be possible to fully understand in the future when experimental data are combined with artificial intelligence methodology.

Information from NMR experiments that have been used to elucidate glycan conformation and dynamics forms the basis for interaction studies between carbohydrate molecules and lectins as well as contributes to the development of small-molecule inhibitors to glycosyl transferases. Furthermore, from NMR experiments it will be possible to gain insight into allosteric regulation of carbohydrate-binding proteins. An understanding of recognition processes where glycans participate may lead to identification of their function in different environments and liquid-state NMR spectroscopy, in conjunction with MD simulations for interpretation of structural data, will definitely contribute to insights on how biomolecular glycan-containing systems operate.
